# Conservation tillage increases carbon sequestration of winter wheat-summer maize farmland on Loess Plateau in China

**DOI:** 10.1371/journal.pone.0199846

**Published:** 2018-09-05

**Authors:** Xingli Lu, Xingneng Lu, Yuncheng Liao

**Affiliations:** 1 College of Agronomy, Ningxia University, Yinchuan, Ningxia, China; 2 Yinchuan Provincial Sub-branch, The People’s Bank of China, Yinchuan, Ningxia, China; 3 College of Agronomy, Northwest A&F University Yangling Shaanxi, China; Tennessee State University, UNITED STATES

## Abstract

The idea of mitigating anthropogenic CO_2_ emissions by increasing soil organic carbon (SOC) is notable. However, the estimation of the net ecosystem carbon balance after conversion from conventional tillage to conservational tillage has been poorly quantified for the Loess Plateau in China. A 2-year field experiment was conducted to estimate the agroecosystem carbon balance of a winter wheat–summer maize rotation system using a full carbon cycle analysis. The results showed that a positive net ecosystem carbon balance value in the cases of rotary tillage with straw incorporation, chisel plow tillage with straw incorporation, and no tillage with straw mulching treatments. Note that a negative value was detected for the conventional moldboard plowing tillage without crop straw treatment. The conversion from conventional tillage to conservational tillage substantially enhanced the carbon sink potential from 0.84 t C ha^−1^ yr^−1^ to 2.69 t C ha^−1^ yr^−1^ in both years. Our findings suggest that the expansion of conservational tillage could enhance the potential carbon sink of the rain-fed land in China.

## Introduction

Agriculture accounts for approximately 10.0%–12.0% of the total global anthropogenic emissions of greenhouse gases (GHGs) [1]. The direct emission of CO_2_ included soil respiration or indirect emission of CO_2_ induced by the production of agriculture inputs (fertilizers and pesticides), fuel combustion, and application of machinery on the farm that is increasing year on year [2]. The winter wheat–summer maize rotation system under a rain-fed condition is one of the major grain productions in North China [3]. Therefore, it is important to study carbon balance in rain-fed fields to select appropriate tillage methods to develop low-carbon agriculture and promote the development of sustainable agriculture.

Previous studies have been conducted to evaluate the carbon source or sink by using several methods such as net carbon flux [4–5], net ecosystem productivity [6], and carbon sustainability [7]. Moreover, previous studies on the carbon balance were primarily focused on forest, grassland, and wetland ecosystems [8–11]. The carbon balance of an agricultural ecosystem is primarily observed in a rice paddy field [12–14]. Conservation tillage treatments (e.g., reduced tillage, no-tillage, and straw returning) are often suggested to improve the potential negative effects of crop residue removal, which may refer to the reduction of soil organic carbon (SOC), the increase in soil compaction, disruption of soil aggregates, and deterioration of soil health [15–17]. Conservation tillage operations have often been reported to enhance soil organic carbon sequestration whereas simultaneously mitigate the carbon (C) emissions associated with agricultural inputs such as fertilizers and on-farm fuels [5]. However, there is considerably uncertainty in the estimation of the carbon sink/source of an agricultural system. For example, Snyder *et al*. [18] has showed that the agricultural fields not only are a carbon sink but also a carbon source because of the application of tillage and fertilizer treatments. Tillage and fertilizer methods always support food, energy, and air for the development of soil organisms, thus increasing the decomposition rate of residues and soil respiration and ultimately resulting in that the stable soil organic carbon is awkward [19]. Li *et al*. [20] measured the carbon balance of winter wheat (−1.98 t hm^−2^) and summer maize (−1.38 t hm^−2^) in the North China Plain, suggesting that without considering the harvest grain carbon part, this ecosystem is a carbon source; however, this ecosystem is a carbon sink when considering the harvest grain carbon part. However, Zhao *et al*. [21] found that the carbon uptake of a farmland in China’s coastal areas is significantly higher than that of C emissions. In addition, Chen *et al*. [22] showed that rotary tillage with straw incorporation and no tillage with straw mulching display a C sink, while moldboard plow tillage with or without straw shows a carbon source in paddy soil. These differences may be attributed to the difference in the level of regional economic development, production layout, and agricultural management practices such as tillage, fertilizer use, and herbicide use.

Soil respiration plays a key role in determining the carbon balance [23]. The lack of available and comprehensive carbon balance data revealed an urgent need to increase the research on the effect of conservation tillage on the net ecosystem carbon balance for the Loess Plateau in China. Thus, the goals of our study are as follows: (1) to estimate the effects of different tillage treatments on soil respiration and its components and (2) to evaluate the effects of different tillage on the net ecosystem carbon balance in the winter wheat–summer maize rotation system.

## Materials and methods

### Study site

**Fig 1:**
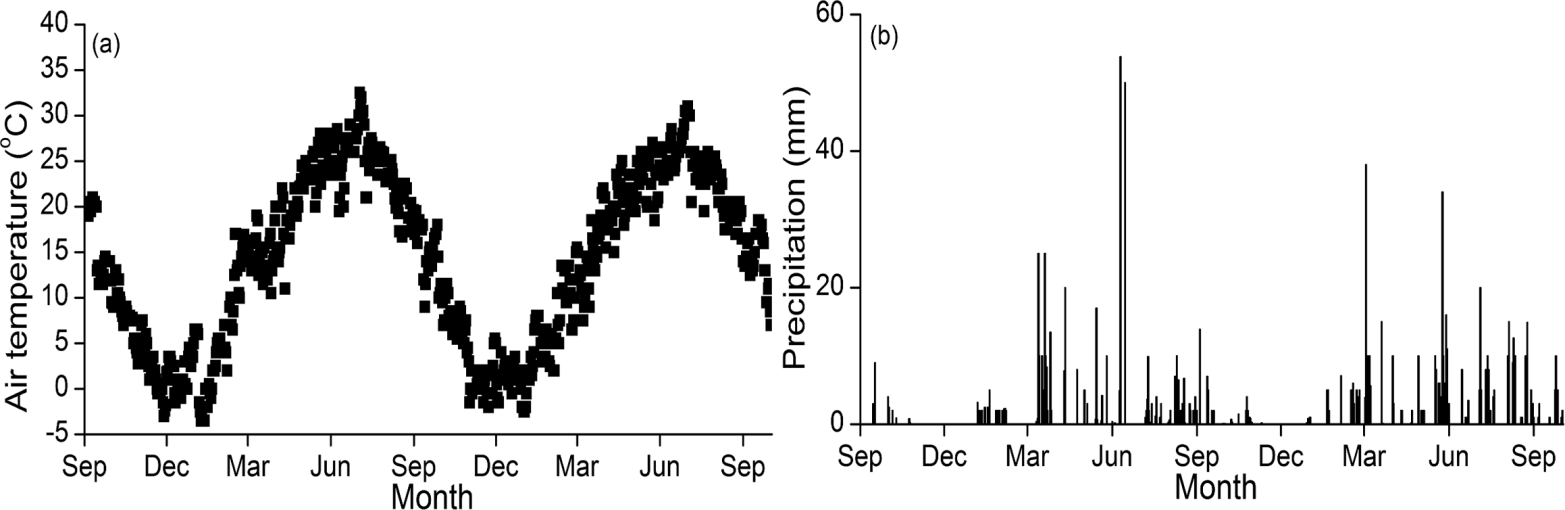
The average of daily air temperature (a) and daily precipitation (b) during the experimental period (from Oct 2013 to Oct 2015).

The study was conducted at the Dry-land Experimental Station of Northwest A&F University, Yangling Town, Shaanxi province, in the northwestern part of China (34°21´N and 108°10´E). The soil is classified as silt loam (19% sand, 77% silt, and 4% clay) based on the USDA Texture Classification System. The surface soil (0–20 cm) bulk density before the start of the experiment (in 2009 year) was 1.30 g cm^-3^. The study area belong to a semiarid climate, and the annual average temperature, the mean annual precipitation, and the annual potential evaporation are 13°C, 622 mm, and 993 mm, respectively. The weather conditions including mean daily air temperature and daily precipitation are presented in Fig 1.

### Experimental design

The experiment had a randomized block design with three replications. Four tillage systems including conventional moldboard plowing tillage without crop straw (CT), rotary tillage with straw incorporation (RTS), chisel plow tillage with straw incorporation (STS), and no tillage with straw mulching (NTS) were included. Thus, 12 plots were designed; the plot size was 48 m^2^ (3.2 m × 15 m). Moreover, three root-free plots with the same size (these were placed at approximately 10-m intervals adjacent to the whole-soil plots) were designed. The root-free plots were established to evaluate microbial respiration (R_h_). The root-free plots were kept free of vegetation by cutting the plants manually throughout the sampling period.

**Fig 2:**
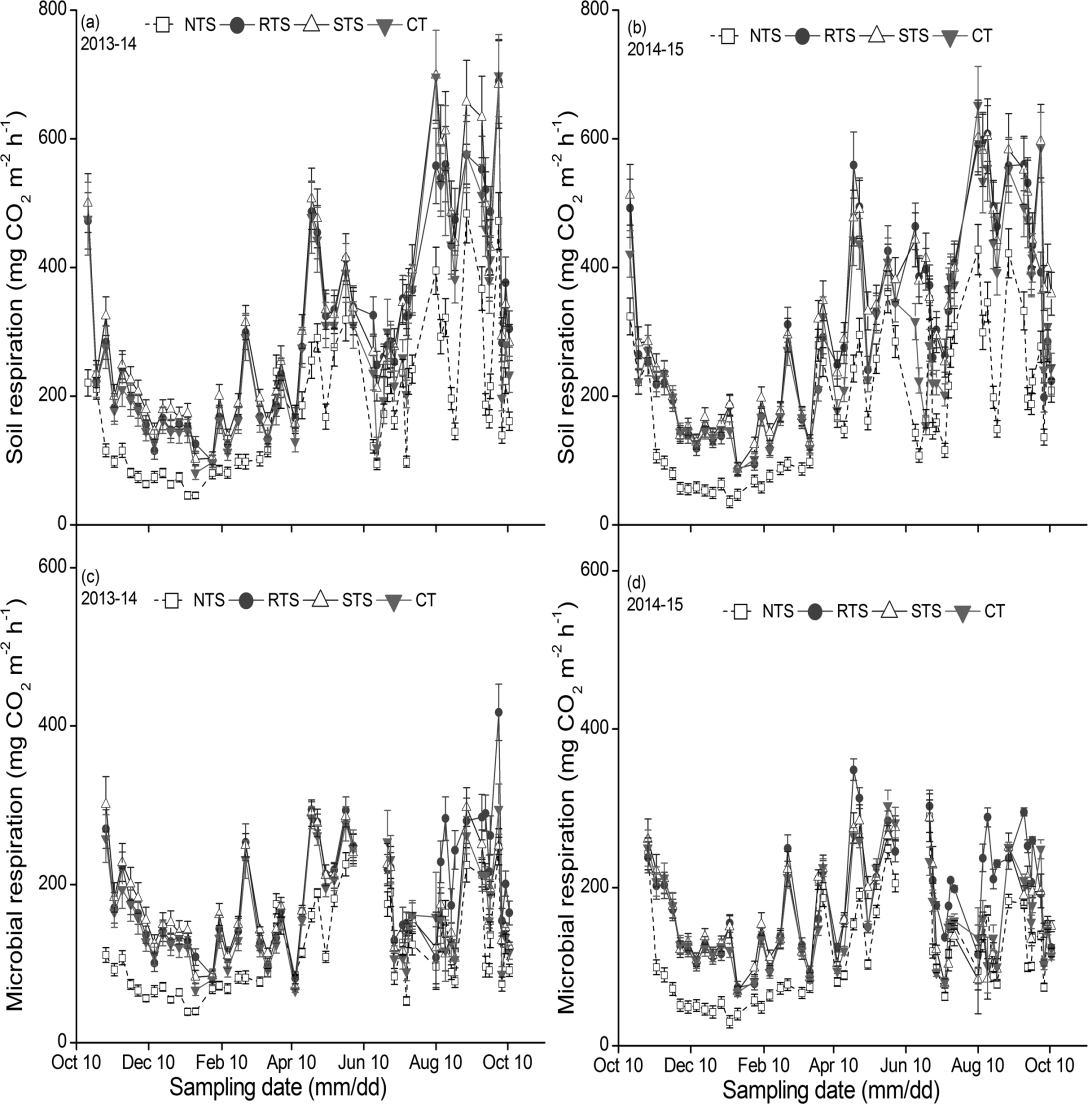
**Season variations of soil respiration (a, 2013–14; b, 2014–15), microbial respiration (c, 2013–14; d, 2014–15) during the cycle of wheat-maize rotation in 2013–2015 in Yangling, China.** (NTS: no tillage with straw mulching; RTS: rotary tillage with straw incorporation; STS: chisel plow tillage with straw incorporation; CT: conventional moldboard plowing tillage without crop straw).

Four tillage treatments, namely, STS, NTS, RTS, and CT, were arranged in three repetitions. The tillage treatments were the same every year since 2009. After harvest, crop straw was removed by hand from the field for the CT treatment before the tillage application. While for the STS, NTS, and RTS treatments, the crop straw was left in the field. The soil tillage was operated twice each year; one was operated on June 17 before planting the summer maize, and the other was operated on October 17 before planting the winter wheat. In the CT plot, the soil was plowed up to a depth of 20–25 cm, and a rotavator was then used to plow the soil up to a depth of 15 cm (Fig 2). In the RTS plot, a rotavator (15 cm) driven by a 95-horsepower tractor (Dong fanghong-LX954, Luoyang, China) was used. In the STS plot, chisel plow machinery was used (30–35 cm). The NTS plots were not disturbed by using the tillage machine either before or after the establishment of the experiment except during sowing with a planter.

### Crop management

Crop cultivation and management were applied in the experiment from October 2013 to October 2015 (Table 1). Winter wheat cultivar Shanmai-139 was sown by using a wheat drill at the rate of 208–210 kg ha^−1^ on October 18, 2013/2014. Summer maize (CV Shandan-609) was planted using a maize drill at the rate of 30 kg ha^−1^ on June 17, 2014/2015. Every year after tillage in June before planting summer maize, all the plots received applications of P_2_O_5_ (172 kg P_2_O_5_ ha^−1^) and N (68 kg N ha^−1^) as the diammonium phosphate fertilizer broadcast. At the seven-leaf stage of summer maize, 172 kg N ha^−1^ was applied as the urea fertilizer by broadcasting according to local recommendations. Similarly, every year after tillage in October, all the plots received applications of P_2_O_5_ (172 kg P_2_O_5_ ha^−1^) and N (68 kg N ha^−1^) as the diammonium phosphate fertilizer broadcast. At the same time, 160 kg N ha^–1^ in the form of urea was applied. Weeds were killed using herbicides. The distance between the rows of wheat was 16 cm. The row spacing was 70 cm, and the plant spacing was 20 cm in the summer maize field. No irrigation was applied at any other time during the entire crop growing season.

**Table 1:** Summary of experimental design with four tillage treatments on Loess Plateau in China.

Type	Operations			
**Soil tillage**	Conventional moldboard plowing tillage	Rotary tillage	Chisel plow tillage	No tillage
**Straw methods**	No straw	Straw incorporation	Straw incorporation	Straw mulching
**Winter crops**	Wheat	Wheat	Wheat	Wheat
**Sowing time**	October	October	October	October
**Harvest time**	June	June	June	June
**Fertilization**	324 kg (NH_4_)_2_HPO_4_ (172 kg P_2_O_5_ ha^-1^; 68 kg N ha^-1^) 348 kg CO(NH_2_)_2_ (160 kg N ha^-1^)	324 kg (NH_4_)_2_HPO_4_ (172 kg P_2_O_5_ ha^-1^; 68 kg N ha^-1^) 348 kg CO(NH_2_)_2_ (160 kg N ha^-1^)	324 kg (NH_4_)_2_HPO_4_ (172 kg P_2_O_5_ ha^-1^; 68 kg N ha^-1^) 348 kg CO(NH_2_)_2_ (160 kg N ha^-1^)	324 kg (NH_4_)_2_HPO_4_ (172 kg P_2_O_5_ ha^-1^; 68 kg N ha^-1^) 348 kg CO(NH_2_)_2_ (160 kg N ha^-1^)
**Summer crops**	Maize	Maize	Maize	Maize
**Sowing time**	June	June	June	June
**Harvest time**	October	October	October	October
**Fertilization**	324 kg (NH_4_)_2_HPO_4_ (172 kg P_2_O_5_ ha^-1^; 68 kg N ha^-1^) 374 kg CO(NH_2_)_2_ (172 kg N ha^-1^)	324 kg (NH_4_)_2_HPO_4_ (172 kg P_2_O_5_ ha^-1^; 68 kg N ha^-1^) 374 kg CO(NH_2_)_2_ (172 kg N ha^-1^)	324 kg (NH_4_)_2_HPO_4_ (172 kg P_2_O_5_ ha^-1^; 68 kg N ha^-1^) 374 kg CO(NH_2_)_2_ (172 kg N ha^-1^)	324 kg (NH_4_)_2_HPO_4_ (172 kg P_2_O_5_ ha^-1^; 68 kg N ha^-1^) 374 kg CO(NH_2_)_2_ (172 kg N ha^-1^)
**Experimental year**	Oct.2013-Oct.2015	Oct.2013-Oct.2015	Oct.2013-Oct.2015	Oct.2013-Oct.2015

### Crop yields and root biomass measurements

At the winter wheat maturity stage, the grain yield of the winter wheat was determined by harvesting three 1-m^2^ sampling areas per treatment by hand. At the summer maize maturity stage, 20 plants from the middle rows per subplot were randomly selected and harvested by hand to determine the yield, and the straw was cut for the crop straw biomass determination. The remaining crop was harvested mechanically. In addition, wheat and corn roots were collected at the ripening stage. To get soil cores, a soil auger (diameter: 8 cm) was applied at three different locations, i.e., at the plant spots, intra-plant spots, and intra-row spots. Each core was taken from a depth of 0 to 100 cm in the soil profile and was incremented by 10 cm, i.e., 0–10, 10–20, 20–30, 30–40, 40–50, 50–60, 60–70, 70–80, 80–90, and 90–100 cm. The soil cores were soaked in a plastic container overnight, and then the root was carefully washed by swirling water through it. The soil material and old dead root debris were separated from the live roots manually. The aboveground and root dry weights were determined after drying the root samples in an oven at 105°C for 30 min and then at 75°C until constant dry weight. After weighing the dry weight, we crushed the aboveground and underground of crops and then, sieved the dry samples by using a 0.25-mm sieve. Then, we placed the samples in clean plastic bags to measure the carbon content. The carbon content of the plant samples was measured using potassium dichromate (K_2_Cr_2_O_4_) and sulfuric acid (H_2_SO_4_) oxidation and ferrous sulfate (FeSO_4_) [24].

### Soil respiration measurements and estimation of root respiration

After planting the crop including winter wheat and summer maize, the PVC chambers (height: 20 cm; inner diameter: 11 cm) were placed and pressed by hand into the soil to a depth of 5 cm for the measurements of R_s_ and R_h_ by the closed chamber method using an infrared gas analyzer (GXH-3010EI, Beijing Huayuan Gas Chemical Industry Co., Ltd., Beijing, China). The samples were placed in field twice each year; one was placed on June 18 after plating the summer maize, and the other was placed on October 20 after planting the winter wheat. The date of measurement of the R_s_ and R_h_ values were shown in S1 and S2 Tables. In each plot, one chamber was located to measure R_s_ in the entire soil. As a result, three chambers were placed in each treatment to measure the R_s_ value in the entire soil for the three replications. Another three chambers were placed in the no-root zone for each root-free plot. The samples were kept free of vegetation by cutting the plants manually throughout the sampling period.

All R_s_ and R_h_ measurements were performed between 9:00 AM and 11:00 AM. local time to avoid the highest CO_2_ emission at noon. The increase in the concentration of CO_2_ within the chamber was measured after three minutes [25]. Root respiration (R_a_) was estimated by subtracting the microbial respiration in the non-root zone from the soil respiration (R_s_) in the whole soil.

The R_s_ was calculated using Eq (1):

$$F=\frac{K(X_{2}-X_{1})H}{\Delta t}$$ (1)

where *F* is the R_s_ value (mg CO_2_ m^−2^ h^−1^); *K* is the reduction coefficient, which is equal to 1.80 at 25°C and 1 Pa; *H* is the height inserted in soil; and $\frac{X_{2}-X_{1}}{\Delta t}$ is the time rate of the change in CO_2_ concentration in the air within the chamber (mg CO_2_ m^−3^ h^−1^). The total R_s_ and its components were calculated as follows:

$$FCO_{2}=\sum_{i}^{n}\frac{F_{i+1}+F_{i}}{2}\times (t_{i+1}‑t_{i})\times 24\times 10^{-4}$$ (2)

Where FCO_2_ is the total emission of CO_2_-C (t ha^-1^), F_i_ is the first CO_2_ emission value (mg CO_2_-C m^-2^ h^-1^) at time t_i_ (h), and F_i+1_ is the following value at time t_i+1_ (h); n is the total number of CO_2_ emission values.

### Measurements of carbon balance

#### System dynamics

The net ecosystem carbon balance from the winter wheat-summer maize production system to the atmosphere was calculated using a full carbon cycle analysis [8–9]. Both carbon fixation within wheat-maize production system and emissions from agricultural practices were considered.

#### Net primary production (NPP)

The NPP (t C ha^−1^) of crops was calculated as follows [26]:

$$NPP=NPP_{grain}+NPP_{straw}+NPP_{root}+NPP_{litter}+NPP_{rhizodeposit}$$ (3)

Grain, straw, and root biomass NPP were converted by applying the dry biomass weight at harvest. Litter was calculated to account for 5% of the aboveground and root dry biomass [27], while rhizodeposits accounted for 18% [28] and 12% [29] of the aboveground and root dry biomass of wheat and maize.

#### Net ecosystem productivity (NEP)

The net ecosystem productivity (NEP) was assessed according to [30] as follows:

$$NEP=NPP-R_{h}$$ (4)

where R_h_ is the microbial respiration measured using the root exclusion technique.

#### Calculation of net ecosystem carbon balance (NCF)

The net ecosystem carbon balance without considering the carbon emission from the farm inputs (NECB) was calculated according to Smith *et al*. [26].

NECB=NEP−Harvest (5)

where NEP is the net ecosystem productivity and harvest means the grain harvest for the STS, NTS, and RTS treatments, while for the CT treatment, harvest means grain + straw.

The net ecosystem carbon balance considering the carbon emission from farm inputs (NCF) was then calculated as follows:

$$NCF=NEP-Harvest-C_{AP}=NECB-C_{AP}$$ (6)

where C_AP_ is the carbon emission from the agricultural input and the data are taken from Lu and Liao [31].

#### Carbon productivity

Carbon productivity (CP) can be calculated by using the following equation [32]:

$$CP=\frac{Y_{c}}{C_{AP}}$$ (7)

where Y_c_ is the grain carbon content (kg C ha^−1^) and C_AP_ is the carbon emission from the agricultural input (kg C ha^−1^).

### Data analysis

Data are shown as the mean values ± standard error. The two-way analysis of variance (ANOVA) with the SAS version 8 software package (SAS Institute, Cary, NC, USA) was used for analyzing the effects of the cropping years and the tillage treatments on the total soil respiration and its components, crop yield, carbon productivity, NPP, NECB, and NCF. When significant, the difference between treatments was determined at the 5% level by applying the least significant difference (LSD) test.

## Results

### Crop production

The grain yields of wheat and maize strongly showed the treatment differences (Table 2). The grain yields ranged from 6.14 to 6.87 t ha^−1^ for wheat and 7.96 to 9.51 t ha^−1^ for maize in 2013–14. No difference in the wheat yield was recorded among the different tillage treatments in 2013–14, while the STS significantly (*p* < 0.05) increased the maize yield by 19.5% as compared with CT. In 2014–15, the STS treatment significantly (*p* < 0.05) increased the wheat yield by 15.4% with respect to CT. No difference in the wheat yield among the NTS, RTS, and CT treatments was recorded. Similar to the 2013–14 cropping year, the STS significantly (*p* < 0.05) increased the maize yield by 20.6% as compared to CT.

**Table 2:** Total soil respiration (R_s_), microbial respiration (R_h_), and wheat and maize grain yields, straw yields and aboveground biomass during the wheat- and maize- growing seasons during the 2013–2015 rotation.

Treatments	Wheat season					Maize season					Annual				
	Rs (t CO_2_-C ha^-1^ yr^-1^)	Rh (t CO_2_-C ha^-1^ yr^-1^)	Grain yield (t ha^-1^)	Straw yield (t ha^-1^)	Aboveground biomass (t ha^-1^)	Rs (t CO_2_-C ha^-1^ yr^-1^)	Rh (t CO_2_-C ha^-1^ yr^-1^)	Grain yield (t ha^-1^)	Straw yield (t ha^-1^)	Aboveground biomass (t ha^-1^)	Rs (t CO_2_-C ha^-1^ yr^-1^)	Rh (t CO_2_-C ha^-1^ yr^-1^)	Grain yield (t ha^-1^)	Straw yield (t ha^-1^)	Aboveground biomass (t ha^-1^)
**2013–14**															
**NTS**	2.02b	1.60c	6.44a	6.50a	12.94ab	2.05b	1.12c	8.20b	8.12ab	16.32b	4.07c	2.72d	14.64b	14.62ab	29.26bc
**RTS**	3.28a	2.58ab	6.36a	6.76a	13.12ab	3.32a	1.70a	8.17b	8.24ab	16.41b	6.60b	4.28a	14.54b	14.99a	29.53b
**STS**	3.51a	2.67a	6.87a	6.56a	13.43a	3.55a	1.46b	9.51a	8.67a	18.18a	7.05a	4.12b	16.38a	15.23a	31.61a
**CT**	3.12a	2.41b	6.14a	6.22a	12.36b	3.17a	1.39b	7.96b	7.73b	15.69b	6.29b	3.80c	14.09b	13.96b	28.05c
**2014–15**															
**NTS**	2.03b	1.60b	6.54ab	6.70a	13.25ab	2.07b	1.11c	8.26b	8.13ab	16.20b	4.10c	2.71c	14.81b	14.83ab	29.44b
**RTS**	3.32a	2.59a	6.36ab	6.27a	12.63b	3.43a	1.79a	8.31b	8.31ab	16.41b	6.75a	4.38a	14.66b	14.58bc	29.04bc
**STS**	3.52a	2.61a	7.20a	6.78a	13.98a	3.56a	1.34b	9.61a	8.87a	18.48a	7.08a	3.95b	16.81a	15.65a	32.46a
**CT**	3.13a	2.42a	6.24b	6.26a	12.49b	3.19a	1.36b	7.97b	7.72b	15.69b	6.32b	3.77b	14.21b	13.97c	28.19c
**LSD0.05**	0.166	0.131	0.267	0.311	0.521	0.283	0.139	0.193	0.423	0.554	0.389	0.213	0.667	0.822	1.244

The different lowercase letters following the same column represent significant difference at 5% levels. CT: conventional moldboard plowing tillage without crop straw; RTS: rotary tillage with straw incorporation; STS: chisel plow tillage with straw incorporation; NTS: no tillage with straw mulching; Rs: soil respiration; Rh: microbial respiration.

### Seasonal variations in R_s_, R_h_, and R_a_

Fig 2 shows the seasonal variation in R_s_ and R_h_ in the 2013–2015 cropping seasons. The seasonal patterns of R_s_ for both seasons showed a similar trend, with the peak appearing in August (at the heading stage of the maize crop) in both the cropping years. After winter wheat was sown, R_s_ was high because of the disturbance of the tillage application. Then, it decreased rapidly and the lowest values during the entire cropping year was recorded at the wintering stage (January 12–19, 2014/2015) because of the low temperature in the winter season. R_s_ then exhibited a dramatic increase when the air temperature recovered and fluctuated till the wheat harvest.

During the maize crop season, R_s_ increased rapidly from July to August and the highest value was recorded at the heading stage (August 10, 2014/2015). The peak value of R_s_ was 395.0 mg CO_2_ m^−2^ h^−1^ for NTS, 557.8 mg CO_2_ m^−2^ h^−1^ for RTS, 698.7 mg CO_2_ m^−2^ h^−1^ for STS, and 696.2 mg CO_2_ m^−2^ h^−1^ for CT in the 2013–14 cropping year. While in the 2014–15 cropping year, the peak value of R_s_ was recorded to be 427.8 mg CO_2_ m^−2^ h^−1^ for NTS, 591.0 mg CO_2_ m^−2^ h^−1^ for RTS, 602.0 mg CO_2_ m^−2^ h^−1^ for STS, and 652.0 mg CO_2_ m^−2^ h^−1 ^for CT. R_s_ then reduced and varied as waves till the maize harvest (Fig 2).

**Fig 3:**
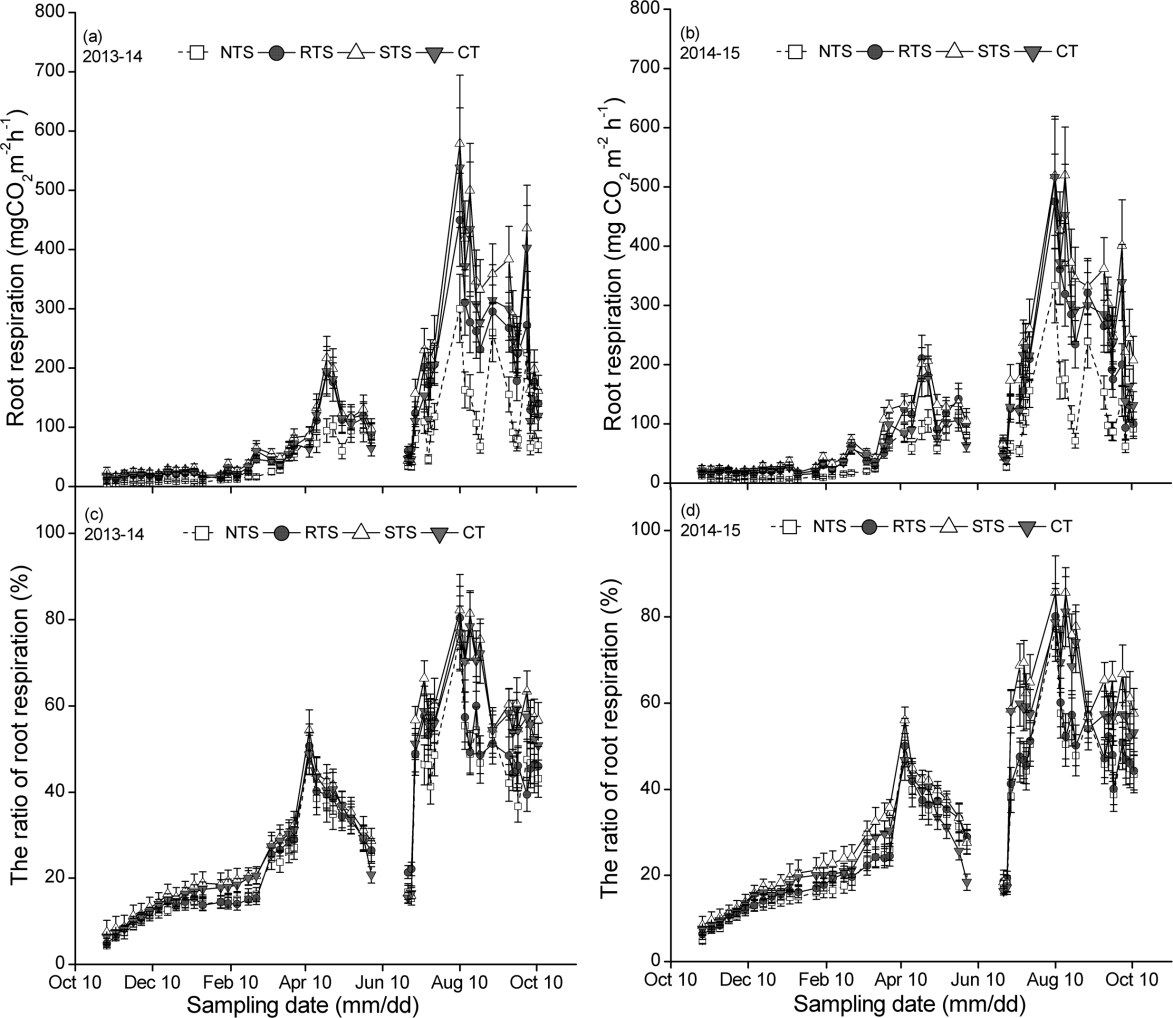
**Season variations of root respiration (a, 2013–14; b, 2014–15) and its ratio (c, 2013–14; d, 2014–15) during the cycle of wheat-maize rotation in 2013–2015 in Yangling, China.** (NTS: no tillage with straw mulching; RTS: rotary tillage with straw incorporation; STS: chisel plow tillage with straw incorporation; CT: conventional moldboard plowing tillage without crop straw).

R_h_ showed a similar trend to that of R_s_ when the crop was small, but a contrasting trend to R_s_ was observed when the crop became big. The R_a_ and R_a_/R_s_ values showed a similar trend, and the season pattern of R_a_ and R_a_/R_s_ was a unimodal curve, corresponding to the growing seasons of wheat and maize (Fig 3). The seasonal pattern of R_a_/R_s_ resembled that of Ra and corresponded clearly to the development of crop (Fig 3). R_a_/R_s_ for wheat exhibited a peak at approximately the flowering stage (April 13–27, 2014/2015). For the maize crop, R_a_/R_s_ exhibited a peak value at approximately the heading stage (August 10–14, 2014/2015). At this time, R_s_ increased and R_h_ decreased, which resulted in an increase in R_a_/R_s_.

### Cumulative R_s_, and R_h_

The cumulative R_s_ emissions from the wheat–maize rotation ranged from 4.07 t C ha^−1^ for NTS to 7.05 t C ha^−1^ for STS (Table 2) in the 2013–14 cropping season. While in the 2014–15 cropping season, the STS treatment significantly (*p* < 0.05) increased the cumulative R_s_ emissions by 12.0% and 72.7% as compared to CT and NTS, respectively. When compared with CT, the NTS significantly (*p* < 0.05) reduced the total R_s_ emissions by 54.1%, no difference in the cumulative R_s_ emissions was found between the RTS and STS treatments (Tables 2 and 3).

**Table 3:** ANOVA of total soil respiration (R_s_), microbial respiration (R_h_), wheat and maize grain yields, straw yields and aboveground biomass during the wheat- and maize- growing seasons during the 2013–2015 rotation.

Effect	d.f.^a^	Wheat season					Maize season					Annual				
		Rs (t CO_2_-C ha^-1^ yr^-1^)	Rh (t CO_2_-C ha^-1^ yr^-1^)	Grain yield (t ha^-1^)	Straw yield (t ha^-1^)	Aboveground biomass (t ha^-1^)	Rs (t CO_2_-C ha^-1^ yr^-1^)	Rh (t CO_2_-C ha^-1^ yr^-1^)	Grain yield (t ha^-1^)	Straw yield (t ha^-1^)	Aboveground biomass (t ha^-1^)	Rs (t CO_2_-C ha^-1^ yr^-1^)	Rh (t CO_2_-C ha^-1^ yr^-1^)	Grain yield (t ha^-1^)	Straw yield (t ha^-1^)	Aboveground biomass (t ha^-1^)
**Block**	2	65.498**	38.379**	19.366**	8.401**	15.748**	19.355**	2.231	92.241**	9.29**	31.017**	41.895**	9.814**	69.687**	23.199**	57.795**
**Year (Y)**	1	0.246	0.108	1.102	0.001	0.268	0.464	0.29	0.758	0.12	0.03	0.5	0.307	1.811	0.098	0.347
**2013–14**		2.98a	2.32a	6.45a	6.51a	12.96a	3.02a	1.42a	8.46a	8.19a	16.65a	6.00a	3.73a	14.91a	14.70a	29.61a
**2014–15**		3.00a	2.31a	6.59a	6.50a	13.09a	3.07a	1.40a	8.54a	8.258a	16.70a	6.07a	3.70a	15.12a	14.76a	29.78a
**LSD0.05**		0.083	0.065	0.267	0.311	0.521	0.142	0.07	0.193	0.423	0.554	0.195	0.106	0.333	0.411	0.622
**Tillage (T)**	3	294.097**	250.871**	8.705**	1.706	4.825*	103.681**	63.449**	63.513**	4.78*	19.666**	216.317**	201.742**	48.218**	9.909**	32.733**
**STS**		3.51a	2.64a	7.04a	6.67a	13.71a	3.55a	1.40b	9.56a	8.77a	18.33a	7.07a	4.04b	16.60a	15.44a	32.04a
**NTS**		2.03d	1.60c	6.49b	6.60a	13.10ab	2.06c	1.11c	8.23bc	8.13b	16.26b	4.09d	2.72d	14.72b	14.73b	29.35b
**RTS**		3.30b	2.59a	6.36b	6.51a	12.87b	3.38ab	1.74a	8.24b	8.27ab	16.41b	6.68b	4.33a	14.59bc	14.79b	29.29b
**CT**		3.13c	2.41b	6.19b	6.24a	12.43b	3.18b	1.37b	7.97c	7.73b	15.69b	6.31c	3.79c	14.16c	13.97c	28.12c
**LSD0.05**		0.117	0.092	0.378	0.439	0.737	0.20	0.098	0.273	0.598	0.783	0.275	0.15	0.471	0.582	0.879
**Y×T**	3	0.033	0.233	0.328	1.319	0.843	0.121	1.71	0.079	0.058	0.12	0.108	1.254	0.229	0.852	0.891
**LSD0.05**		0.166	0.131	0.535	0.311	1.042	0.283	0.139	0.386	0.846	1.108	0.195	0.213	0.667	0.822	1.244

The different lowercase letters following the same column represent significant difference at 5% levels

** is significant at the P≤0.01 level

* is significant at the P≤0.05 level.

CT: conventional moldboard plowing tillage without crop straw; RTS: rotary tillage with straw incorporation; STS: chisel plow tillage with straw incorporation; NTS: no tillage with straw mulching; Rs: soil respiration; Rh: microbial respiration.

Similarly, the lowest cumulative R_h_ emissions were recorded in the NTS treatment. The NTS significantly (*p* < 0.05) reduced the cumulative R_h_ emissions from 28.4% to 36.4% as compared to the other three treatments in the 2013–14 cropping season. While in the 2014–15 cropping season, this reduction percentage varied from 28.1% to 38.1% (Table 2).

### Net ecosystem carbon balance under conservation tillage treatments

The calculations of the NCF from the estimates of the potential carbon inputs from the aboveground biomass, root biomass, negative cumulative carbon loss via R_h_, and agricultural input emissions resulted in differences among the different tillage treatments. The NPP values ranged from 10.4 to 11.7 t C ha^−1^ yr^−1^ in 2013–14 and from 10.5 to 12.0 t C ha^−1^ yr^−1^ in 2014–15 (Table 4). In the 2013–14 and 2014–15 cropping years, the carbon loss through R_h_ in the winter wheat–summer maize ecosystem accounted for NPP 25.1%–39.1% and 24.7%–40.4%, and the harvest part accounted for NPP 28.8%–90.5% and 45.6%–90.4%.

**Table 4:** The agro-ecosystem C balance (NCF) and its main components for the annual cycle of wheat-maize rotation in 2013–2015 (t C ha^-1^ yr^-1^).

Year	Treatment	NPP	R_h_	Harvest	NECB	C_AP_	NCF
	NTS	10.85bc	2.72d	4.95c	3.12a	0.59	2.53a
**2013–14**	RTS	10.95b	4.28a	4.91c	1.72b	0.67	1.05b
	STS	11.71a	4.12b	5.53b	1.91b	0.67	1.24b
	CT	10.40c	3.80c	9.41a	-2.85c	0.76	-3.61c
	NTS	10.99b	2.71c	5.01c	3.28a	0.59	2.69a
**2014–15**	RTS	10.84bc	4.38a	4.95c	1.51c	0.67	0.84c
	STS	12.02a	3.95b	5.68b	2.39b	0.67	1.72b
	CT	10.45c	3.77b	9.45a	-2.77d	0.76	-3.53d
**LSD0.05**		0.461	0.213	0.292	0.390		0.390

The different lowercase letters following the same column represent significant difference at 5% levels. CT: conventional moldboard plowing tillage without crop straw; RTS: rotary tillage with straw incorporation; STS: chisel plow tillage with straw incorporation; NTS: no tillage with straw mulching; NPP: net primary productivity; Rh: microbial respiration; NECB: net ecosystem carbon balance without considering the carbon emission from farm inputs; C_AP_: the carbon emission from agricultural input; NCF: the net ecosystem carbon balance with considering the carbon emission from farm inputs.

The overall NCFs were significantly (*p* < 0.05) affected by the tillage practices (Tables 4 and 5). The lowest NCF value was found under the conventional moldboard plow tillage treatment in the rain-fed winter wheat–summer maize field on the Loess Plateau in China at −3.61 t C ha^−1^ yr^−1^ in 2013–14, and −3.53 t C ha^−1^ yr^−1^ in 2014–15, whereas the highest NCF value was found under NTS at 2.53 t C ha^−1^ yr^−1^ in 2013–14, and 2.69 t C ha^−1^ yr^−1^ in 2014–15. This may be attributed to the lower carbon loss via R_h_ and higher carbon returning in the NTS treatment. There was no significant difference in the NCF values between the RTS and the STS treatments. Over the cycle of wheat–maize rotation, all of the treatments, except for the CT, led to carbon gains of 1.05 to 2.53 t C ha^−1^ yr^−1^ in 2013–14, and 0.84 to 2.69 t C ha^−1^ yr^−1^ in 2014–15. The CT treatment had a negative NCF value, mainly attributed to the higher harvest part as compared to the other straw returning treatments. The carbon in an agroecosystem mainly depended on the addition of organic matter; in the present study, positive NCF under three conservation tillage treatments was mainly attributed to the amount of crop residues in the soil. These results pointed out the importance of using crop straw in no tillage and reduced tillage for increasing the carbon input in the wheat–maize field on the Loess Plateau in China.

**Table 5:** ANOVA of net primary productivity (NPP), harvest, net ecosystem carbon balance and net carbon flux during the wheat- and maize- growing seasons during the 2013–2015 rotation.

Effect	D.f.^a^	NPP	Harvest	NECB	NCF
**Block**	2	57.912**	59.387**	28.738**	28.738**
**Year (Y)**	1	0.866	1.135	1.966	1.966
**2013–14**		10.98a	6.201a	0.974a	0.302a
**2014–15**		11.08a	6.273a	1.102a	0.429a
**LSD0.05**		0.231	0.146	0.195	0.195
**Tillage (T)**	3	31.523**	998.385**	848.846**	891.298**
**STS**		11.87a	5.607b	2.15b	1.48b
**NTS**		10.92b	4.978c	3.20a	2.61a
**RTS**		10.90b	4.933c	1.61c	0.94c
**CT**		10.43c	9.430a	-2.81d	-3.57d
**LSD0.05**		0.326	0.206	0.276	0.276
**Y×T**	3	0.688	0.134	2.458	2.458
**LSD0.05**		0.461	0.292	0.390	0.390

The different lowercase letters following the same column represent significant difference at 5% levels

** is significant at the P≤0.01 level.

CT: conventional moldboard plowing tillage without crop straw; RTS: rotary tillage with straw incorporation; STS: chisel plow tillage with straw incorporation; NTS: no tillage with straw mulching; NPP: net primary productivity; Rh: microbial respiration; NECB: net ecosystem carbon balance without considering the carbon emission from farm inputs; CAP: the carbon emission from agricultural input; NCF: the net ecosystem carbon balance with considering the carbon emission from farm inputs.

### Effect of conservation tillage on carbon productivity

**Fig 4:**
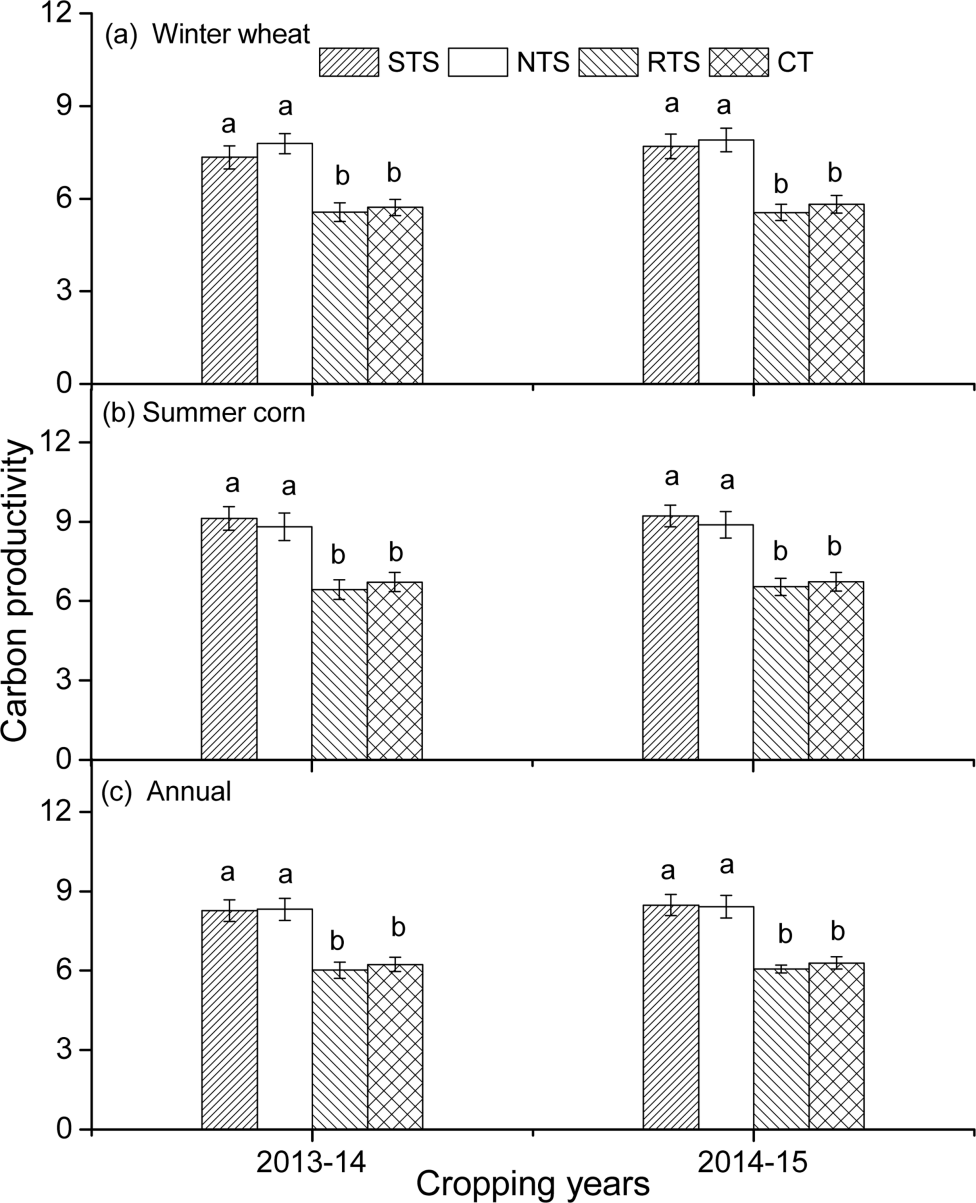
**Effect of different tillage treatments on carbon productivity of winter wheat (a), summer corn (b), and annual (c) in both cropping years.** (The different lowercase letters above the error bars represent significant difference between different tillage treatments within a two-year period at 5% levels according to the LSD test. CT: conventional moldboard plowing tillage without crop straw; RTS: rotary tillage with straw incorporation; STS: chisel plow tillage with straw incorporation; NTS: no tillage with straw mulching).

Tillage significantly (*p* < 0.05) affected the carbon productivity of the winter wheat (Fig 4). When compared with CT, STS and NTS significantly increased the carbon productivity of winter wheat by 28.3% and 36.0% in 2013–14, respectively. This increase percentage was 32.4% and 36.0% in the 2014–15 cropping year. Similarly, STS and NTS significantly improved the carbon productivity of summer corn from 31.1% to 36.8% in both the years. When compared with the CT treatment, STS, NTS, and RTS significantly increased the annual carbon productivity by 32.4%, 33.3%, and 17.6% in 2013–14, while in 2014–15, STS, NTS, and RTS significantly increased the annual carbon productivity by 34.6%, 33.7%, and 17.5% as compared to the CT treatment.

## Discussion

### Effect of conservation tillage on crop yields

In the present study, STS significantly increased the wheat yield by 15.4% as compared to the CT treatment in the 2014–15 cropping year. For the maize crop, when compared with CT, the STS treatment significantly improved the maize yield by 19.5% in 2013–14 and 20.6% in 2014–15, respectively. Similarly, Wang *et al*. [33] reported that chisel plow tillage is beneficial for reducing the soil bulk density, improving soil structure, increasing soil water availability, and aeration. Xu *et al*. [34] also reported that when compared to conventional tillage, chisel plow could improve the yield of crops by loosening the soil and promoting the root growth of the crop. In the present study, no difference in the grain yield was recorded between the NTS and the CT treatments. However, Liu *et al*. [35] reported that no tillage is beneficial for improving the crop yield and water use efficiency in Weibei Highland, China. These inconsistent results may be attributed to the difference in the cropping years, crop, climate conditions, and the duration of conservation tillage. Our results suggest that long-term studies are required to identify the initial and long-term yield constraints of conservation tillage.

### Effect of conservation tillage on R_s_ and its components

The two-year data showed that NTS reduced R_s_ by 13.0% as compared to the other tillage treatments, which was mainly attributed to the lack of disturbance in NTS after the harvest of summer maize. In addition, when compared with CT, the STS and RTS treatments increased the total annual R_s_ by 12.0% and 6.8%, respectively. This was mainly attributed to the organic carbon input and the tillage disturbance. Li *et al*. [36] also reported that R_s_ increases when the organic carbon is input into the soil. Our results suggested that the organic carbon input in the STS, RTSm and NTS treatments promoted the activity of microbial and ultimately affected the decomposition of the organic matter and the release of soil CO_2_, and influenced the carbon balance. Although STS and RTS increased the R_h_ value, the less carbon input (i.e., diesel fuel and the carbon loss by residue removal) compensated for the higher carbon emissions; finally, STS and RTS showed the carbon sink. In addition, the highest R_h_ value was recorded in RTS, which was mainly attributed to the fact that the tillage depth was only 15 cm, which made it easier for the microbes to touch the residue.

### NCF in winter wheat-summer corn ecosystem under different tillage treatments

This is a very important method to mitigate the atmospheric CO_2_ emission by increasing the soil carbon pool [18]. This study focused on the effect of different tillage treatments on the carbon balance of the winter wheat–summer maize ecosystem. The carbon storage or loss mainly relied on the balance of carbon fixed into the soil through the addition of residues and carbon loss through the respiration in a dryland agricultural ecosystem. Our results showed that the carbon loss through R_h_ and agricultural inputs accounted for NPP 24.7%–40.4% in the different tillage treatments in the 2013–14 and 2014–15 cropping years, respectively. In addition, conservation tillage practices such as the NTS, RTS, and STS treatments were beneficial for carbon sequestration irrespective of the carbon input from the agricultural inputs, which agreed with the other reported results [37–39]. Moreover, the contribution of the agricultural input to the total carbon emission varied from 5.1% to 17.8% in the different tillage treatments, which indicated that the carbon emission from the agricultural input should also be included when evaluating the carbon sink.

Moreover, the positive value of NCF was mainly attributed to the large amount input of the crop straws in the three conservation tillage treatments. In the experiment, the carbon input under the NTS, RTS, and STS treatments varied from 5.90–6.34 t C ha^-1^ yr^-1^ in both years. Han *et al*. [40] also showed that SOC sequestration increases with the annual input rate of straw C input rate. The winter wheat–summer maize ecosystem showed a carbon sink for all the treatments without considering the carbon harvest part. However, when the carbon harvest part was derived from NPP − R_h_, this ecosystem was a carbon source for the CT treatment. Our results were similar to those of Li *et al*. [41] who showed that the winter wheat–summer maize rotation ecosystem was a carbon sink without considering the harvest carbon part; however, the carbon sink changed into a source when the harvest carbon part was included. Moreover, when considering the carbon emission from the agricultural input, the carbon balance decreased for all the treatments. Thus, our results suggested that the purpose of the carbon sink could be realized by increasing the carbon inputs of the crop residues and roots.

### Carbon productivity

When combining the winter wheat and the summer maize, the highest annual carbon productivity was observed in the STS and NTS treatments, and the lowest value in the CT treatment. In the NTS treatment, the higher carbon productivity was mainly attributed to the lower carbon emission from the farm input because of the similar grain yield to that in the CT treatment. While for the STS treatment, the higher carbon productivity was mainly attributed to the higher crop productivity. Similarly, van den Putte *et al*. [42] reported that deep conservation tillage performs better than superficial conservation tillage. This indicated that plants could benefit from the increased pore space and aeration at deeper depths. In addition, studies have already shown that the root growth conditions for cereals are less favorable in the case of the NT treatment than in the case of the CT treatment [43–44]. However, Boomsma *et al*. [45] showed that as compared to the moldboard plow, conservation tillage reduced the crop yields. This was mainly attributed to the relatively poor seedbed conditions, delayed seedling emergence, and crop development in the case of conservation tillage with respect to those in the case of the moldboard plow tillage treatment. Chisel plow tillage may therefore be expected to be more favorable than superficial tillage for crops. Our results also suggested that the conversion of treatment from conventional tillage to conservation tillage could increase the annual carbon productivity.

## Conclusions

Our results showed that heterotrophic (microbial) respiration was lower in the NTS treatment than in other three tillage treatments. In the case of the CT treatment in the winter wheat–summer maize field on China’s Loess Plateau, carbon added as the aboveground biomass and root biomass was not sufficient to compensate for the loss of carbon from organic matter decomposition, rendering the rain-fed winter wheat–summer maize field as the net sources of atmospheric CO_2_. The conversion from conventional tillage to conservational tillage substantially enhanced the carbon sink potential from 0.84 t C ha^−1^ yr^−1^ in RTS to 2.69 t C ha^−1^ yr^−1^ in the NTS treatment. Thus, the expansion of conservational tillage could enhance the potential carbon sink of rain-fed land in China’s Loess Plateau. Our results also showed the importance of the returning of crop straw to the field in order to change the winter wheat–summer maize ecosystem from carbon source to sink.

## Supporting information

S1 Table Soil respiration under different tillage treatments. (DOCX)

S2 Table Soil microbial respiration under different tillage treatments. (DOCX)

S3 Table Root Respiration under different tillage treatments. (DOCX)

S4 Table The ratio of root respiration to total soil respiration under different tillage treatments. (DOCX)

S5 Table The total respiration (R_s_), microbial respiration (R_h_), wheat and maize grain yields, straw yields and aboveground biomass during the wheat- and maize- growing seasons during the 2013–2015 rotation. (DOCX)

S6 Table The agro-ecosystem C balance (NCF) and its main components for the annual cycle of wheat-maize rotation in 2013–2015. (DOCX)

S7 Table The carbon productivity under different treatments. (DOCX)
