# Supplementary material for: Conservation tillage increases carbon sequestration of winter wheat-summer maize farmland on Loess Plateau in China
Source: PLoS One. 2018 Sep 5;13(9):e0199846. doi: 10.1371/journal.pone.0199846 (PMC6124710; doi:10.1371/journal.pone.0199846)
Supplement: S1 Table — (DOCX) [file pone.0199846.s001.docx]

**S1 Table. Soil respiration under different tillage treatments (****mg CO_2_ m^−2^ h^−1^).**

| **Sampling date** | **NTS** | **SE** | **RTS** | **SE** | **STS** | **SE** | **CT** | **SE** |
| --- | --- | --- | --- | --- | --- | --- | --- | --- |
| 2013/10/20 | 220.58 | 20.35 | 472.3 | 43.44 | 500.2 | 45.85 | 475.4 | 56.72 |
| 2013/10/27 | 211.83 | 16.69 | 223.4 | 25.21 | 234.5 | 20.85 | 218.7 | 20.27 |
| 2013/11/4 | 115.6 | 10.09 | 283.4 | 26.20 | 324.5 | 29.90 | 275.4 | 32.85 |
| 2013/11/11 | 98.11 | 8.75 | 182.3 | 21.18 | 200.3 | 17.35 | 178.2 | 22.00 |
| 2013/11/18 | 115.6 | 10.70 | 243 | 21.67 | 248.7 | 21.68 | 210.6 | 27.16 |
| 2013/11/25 | 80.61 | 7.27 | 198 | 18.88 | 225.6 | 19.81 | 194.4 | 25.61 |
| 2013/12/1 | 73.92 | 8.57 | 183.2 | 17.41 | 206.5 | 18.19 | 178.2 | 25.06 |
| 2013/12/8 | 63.12 | 5.47 | 155.3 | 13.97 | 178.6 | 15.74 | 145.8 | 15.52 |
| 2013/12/15 | 73.92 | 8.57 | 115.6 | 13.32 | 145.6 | 12.90 | 129.6 | 18.08 |
| 2013/12/22 | 80.61 | 7.27 | 165.4 | 17.63 | 178.6 | 15.74 | 162 | 20.22 |
| 2013/12/29 | 63.12 | 5.59 | 148 | 13.91 | 179.6 | 15.68 | 145.8 | 18.48 |
| 2014/1/5 | 73.92 | 6.95 | 156.7 | 8.42 | 167.8 | 15.18 | 144.8 | 18.22 |
| 2014/1/12 | 45.62 | 5.83 | 152.2 | 14.33 | 173.4 | 15.08 | 145.8 | 18.48 |
| 2014/1/19 | 45.62 | 5.14 | 125.4 | 11.34 | 102.3 | 9.39 | 81 | 10.54 |
| 2014/2/2 | 80.61 | 9.23 | 98.7 | 9.04 | 103.4 | 10.17 | 97.2 | 11.73 |
| 2014/2/8 | 84.72 | 7.67 | 167.8 | 17.86 | 200.3 | 17.35 | 162 | 19.78 |
| 2014/2/15 | 80.61 | 8.60 | 124.5 | 14.19 | 136.7 | 12.25 | 113.4 | 13.63 |
| 2014/2/24 | 98.11 | 10.95 | 165.7 | 17.66 | 187.6 | 16.26 | 162 | 19.78 |
| 2014/3/2 | 98.11 | 11.61 | 298.7 | 26.78 | 314.5 | 26.45 | 291.6 | 36.61 |
| 2014/3/14 | 102.21 | 10.76 | 168.9 | 15.03 | 197.6 | 12.76 | 164.8 | 20.92 |
| 2014/3/21 | 115.6 | 10.04 | 134.2 | 12.54 | 160.4 | 14.04 | 129.6 | 17.09 |
| 2014/3/28 | 238.08 | 26.04 | 185.6 | 16.92 | 205.6 | 17.81 | 178.2 | 22.52 |
| 2014/4/1 | 203.08 | 21.96 | 234.4 | 25.07 | 254.6 | 23.36 | 226.8 | 28.61 |
| 2014/4/13 | 168.09 | 17.89 | 165.4 | 16.06 | 156.7 | 14.77 | 129.6 | 16.52 |
| 2014/4/19 | 185.59 | 20.85 | 276.5 | 25.88 | 300.5 | 26.50 | 275.4 | 30.85 |
| 2014/4/27 | 255.57 | 28.39 | 486.5 | 46.35 | 506.7 | 47.56 | 478.2 | 56.23 |
| 2014/5/2 | 290.56 | 21.99 | 454.6 | 41.38 | 476.5 | 45.78 | 443 | 50.23 |
| 2014/5/9 | 168.09 | 19.79 | 324.5 | 34.62 | 334.5 | 30.56 | 310.6 | 34.94 |
| 2014/5/16 | 276.09 | 29.22 | 334.5 | 30.56 | 326.7 | 29.67 | 310.6 | 35.41 |
| 2014/5/26 | 319.51 | 34.13 | 413.2 | 39.47 | 415.6 | 36.72 | 391.6 | 45.71 |
| 2014/6/1 | 331.6 | 36.53 | 339.2 | 32.52 | 336.7 | 26.53 | 310.6 | 36.41 |
| 2014/6/18 | 122 | 10.57 | 325.6 | 29.00 | 269 | 24.78 | 235.9792 | 26.44 |
| 2014/6/21 | 94 | 8.24 | 248.7 | 20.60 | 212 | 19.28 | 119.9943 | 16.34 |
| 2014/6/27 | 172.4 | 15.16 | 261 | 19.76 | 254 | 23.31 | 192.9909 | 35.99 |
| 2014/6/30 | 210 | 18.73 | 278.9 | 24.41 | 269 | 26.90 | 299.9957 | 50.57 |
| 2014/7/3 | 224 | 20.08 | 284.3 | 25.78 | 263 | 26.30 | 277.0187 | 39.12 |
| 2014/7/6 | 163 | 14.28 | 253 | 23.57 | 277 | 27.70 | 216.0153 | 23.24 |
| 2014/7/13 | 236 | 21.25 | 351 | 37.01 | 346 | 34.60 | 259.9793 | 27.18 |
| 2014/7/16 | 98 | 8.56 | 324 | 29.40 | 339 | 33.90 | 202.9904 | 21.43 |
| 2014/7/18 | 223 | 19.99 | 325.6 | 36.78 | 362 | 36.20 | 350.0014 | 36.55 |
| 2014/7/21 | 242 | 21.83 | 364.5 | 38.84 | 396 | 39.60 | 367.018 | 37.50 |
| 2014/8/10 | 395 | 36.87 | 557.8 | 58.44 | 698.7 | 69.87 | 696.2172 | 72.33 |
| 2014/8/14 | 292 | 26.71 | 538.9 | 51.15 | 593 | 59.30 | 528.2134 | 48.89 |
| 2014/8/18 | 322 | 29.66 | 560.4 | 58.47 | 612 | 61.20 | 550.39 | 101.23 |
| 2014/8/23 | 196 | 17.39 | 434.5 | 44.90 | 485.4 | 48.54 | 435.204 | 52.94 |
| 2014/8/26 | 145 | 12.62 | 474.3 | 48.80 | 438.7 | 43.87 | 382.202 | 36.98 |
| 2014/9/5 | 484 | 45.70 | 575.2 | 51.22 | 656.7 | 65.67 | 575.9936 | 60.26 |
| 2014/9/18 | 367 | 34.10 | 552.3 | 51.36 | 633.4 | 63.34 | 511.9978 | 48.42 |
| 2014/9/21 | 187 | 16.54 | 521.3 | 49.39 | 498.6 | 49.86 | 460.0206 | 43.18 |
| 2014/9/24 | 164.5 | 14.42 | 393.5 | 45.56 | 392.3 | 39.23 | 380.0171 | 51.84 |
| 2014/9/25 | 215 | 19.22 | 486.5 | 39.56 | 431.3 | 43.13 | 412.0183 | 37.70 |
| 2014/10/2 | 472 | 44.50 | 689.4 | 65.33 | 684.3 | 68.43 | 697.9921 | 63.79 |
| 2014/10/5 | 139 | 12.08 | 283.2 | 30.33 | 293 | 29.30 | 196.9977 | 20.29 |
| 2014/10/8 | 224 | 20.08 | 376.5 | 39.74 | 343.2 | 34.32 | 280.0085 | 25.78 |
| 2014/10/11 | 161 | 14.09 | 304.3 | 28.00 | 283.5 | 28.35 | 233.545 | 28.14 |
| 2014/10/20 | 324.0 | 28.74 | 492.0 | 45.04 | 512.8 | 47.08 | 421.0 | 36.47 |
| 2014/10/27 | 222.0 | 19.26 | 265.0 | 23.18 | 283.0 | 24.85 | 223.2 | 19.36 |
| 2014/11/4 | 254.0 | 22.17 | 254.0 | 22.17 | 285.0 | 25.04 | 270.3 | 23.65 |
| 2014/11/11 | 107.0 | 10.37 | 218.0 | 18.90 | 244.0 | 21.25 | 226.5 | 19.68 |
| 2014/11/18 | 96.7 | 9.84 | 221.0 | 19.17 | 235.0 | 20.43 | 234.0 | 20.36 |
| 2014/11/25 | 79.4 | 9.15 | 193.0 | 16.72 | 199.0 | 17.24 | 198.6 | 17.31 |
| 2014/12/1 | 56.7 | 8.69 | 142.0 | 12.63 | 139.0 | 12.42 | 145.0 | 12.64 |
| 2014/12/8 | 55.0 | 8.67 | 139.0 | 12.42 | 152.0 | 13.38 | 146.0 | 12.91 |
| 2014/12/15 | 58.0 | 8.70 | 119.0 | 11.08 | 129.0 | 11.72 | 127.0 | 11.58 |
| 2014/12/22 | 53.0 | 8.67 | 149.0 | 13.16 | 166.9 | 14.55 | 145.8 | 12.91 |
| 2014/12/29 | 49.2 | 8.66 | 132.0 | 11.93 | 138.0 | 12.35 | 136.0 | 12.20 |
| 2015/1/5 | 63.4 | 8.76 | 138.0 | 12.35 | 158.0 | 13.84 | 149.0 | 13.14 |
| 2015/1/12 | 35.6 | 8.78 | 185.0 | 16.04 | 186.7 | 16.18 | 147.0 | 13.00 |
| 2015/1/19 | 46.8 | 8.67 | 88.0 | 9.46 | 86.9 | 9.41 | 83.6 | 7.91 |
| 2015/2/2 | 68.3 | 8.85 | 94.0 | 9.72 | 125.0 | 11.46 | 102.0 | 8.81 |
| 2015/2/8 | 58.0 | 8.70 | 169.0 | 14.71 | 197.6 | 17.11 | 168.0 | 14.64 |
| 2015/2/15 | 76.0 | 9.04 | 119.0 | 11.07 | 144.0 | 12.78 | 116.0 | 10.89 |
| 2015/2/24 | 88.0 | 9.46 | 174.0 | 15.12 | 176.0 | 15.29 | 168.7 | 14.72 |
| 2015/3/2 | 94.8 | 9.75 | 311.0 | 27.50 | 294.8 | 25.97 | 271.0 | 23.74 |
| 2015/3/14 | 86.9 | 9.41 | 164.0 | 14.32 | 169.0 | 14.72 | 166.8 | 14.83 |
| 2015/3/21 | 98.4 | 9.92 | 123.0 | 11.33 | 128.0 | 11.66 | 119.0 | 10.37 |
| 2015/3/28 | 279.2 | 24.50 | 210.6 | 18.25 | 320.0 | 28.35 | 210.6 | 18.25 |
| 2015/4/1 | 253.0 | 22.07 | 291.6 | 25.66 | 348.2 | 31.06 | 324.0 | 29.86 |
| 2015/4/13 | 165.8 | 14.46 | 248.2 | 21.63 | 235.8 | 20.50 | 178.2 | 15.73 |
| 2015/4/19 | 148.0 | 13.08 | 275.0 | 24.11 | 289.0 | 25.42 | 210.0 | 18.19 |
| 2015/4/27 | 243.0 | 21.15 | 559.2 | 51.65 | 476.8 | 43.55 | 443.0 | 40.20 |
| 2015/5/2 | 295.0 | 25.99 | 494.0 | 45.24 | 490.0 | 44.85 | 437.0 | 39.66 |
| 2015/5/9 | 162.0 | 14.16 | 240.6 | 20.94 | 332.0 | 29.50 | 225.8 | 19.60 |
| 2015/5/16 | 258.0 | 22.53 | 331.0 | 29.40 | 342.0 | 30.46 | 327.0 | 29.03 |
| 2015/5/26 | 362.0 | 32.38 | 426.0 | 38.59 | 400.0 | 36.05 | 409.0 | 36.93 |
| 2015/6/1 | 284.8 | 25.02 | 345.8 | 30.82 | 381.0 | 34.21 | 345.8 | 30.84 |
| 2015/6/18 | 143.2 | 12.72 | 464.0 | 35.91 | 443.0 | 42.02 | 316.0 | 27.97 |
| 2015/6/21 | 108.0 | 10.42 | 386.0 | 31.93 | 379.0 | 35.66 | 224.0 | 19.44 |
| 2015/6/27 | 165.0 | 14.40 | 397.0 | 32.48 | 414.2 | 39.16 | 154.0 | 13.53 |
| 2015/6/30 | 350.3 | 31.25 | 372.4 | 31.25 | 353.5 | 33.13 | 279.0 | 24.48 |
| 2015/7/3 | 146.0 | 12.93 | 259.7 | 25.75 | 279.4 | 25.81 | 221.0 | 19.17 |
| 2015/7/6 | 158.7 | 13.90 | 303.2 | 27.83 | 294.6 | 27.31 | 221.0 | 19.17 |
| 2015/7/13 | 116.0 | 10.89 | 263.0 | 25.91 | 253.0 | 23.21 | 202.0 | 17.49 |
| 2015/7/16 | 215.8 | 18.71 | 332.0 | 29.24 | 340.0 | 31.80 | 366.0 | 32.77 |
| 2015/7/18 | 268.0 | 23.46 | 384.0 | 31.83 | 371.0 | 34.87 | 386.0 | 34.70 |
| 2015/7/21 | 308.9 | 27.30 | 408.0 | 33.04 | 399.0 | 37.65 | 373.0 | 33.44 |
| 2015/8/10 | 427.8 | 38.76 | 591.0 | 42.54 | 602.0 | 57.86 | 652.0 | 60.82 |
| 2015/8/14 | 299.0 | 26.36 | 598.0 | 42.91 | 581.8 | 55.85 | 534.0 | 49.17 |
| 2015/8/18 | 346.0 | 30.84 | 608.0 | 43.44 | 604.0 | 58.06 | 554.0 | 51.14 |
| 2015/8/23 | 198.0 | 17.15 | 495.0 | 37.51 | 489.0 | 46.60 | 438.0 | 39.75 |
| 2015/8/26 | 149.0 | 13.15 | 464.0 | 35.91 | 442.0 | 41.92 | 392.0 | 35.28 |
| 2015/9/5 | 422.0 | 38.19 | 558.0 | 40.80 | 583.0 | 55.97 | 551.0 | 50.84 |
| 2015/9/18 | 332.0 | 29.50 | 560.0 | 40.91 | 551.0 | 52.78 | 493.0 | 45.14 |
| 2015/9/21 | 196.0 | 16.98 | 531.0 | 39.39 | 517.0 | 49.39 | 474.0 | 43.28 |
| 2015/9/24 | 188.0 | 16.29 | 398.0 | 32.53 | 394.0 | 37.15 | 389.0 | 34.99 |
| 2015/9/25 | 223.0 | 19.35 | 434.0 | 34.37 | 443.0 | 42.02 | 415.0 | 37.51 |
| 2015/10/2 | 277.0 | 24.30 | 392.0 | 32.23 | 596.0 | 57.26 | 587.0 | 54.39 |
| 2015/10/5 | 136.0 | 12.20 | 198.0 | 22.92 | 368.0 | 34.57 | 241.0 | 20.97 |
| 2015/10/8 | 278.0 | 24.39 | 285.0 | 26.95 | 399.0 | 37.65 | 309.0 | 27.31 |
| 2015/10/11 | 209.0 | 18.11 | 224.0 | 24.09 | 359.0 | 33.68 | 245.0 | 21.34 |

CT, conventional moldboard plowing tillage without crop straw; RTS, rotary tillage with straw incorporation; STS, chisel plow tillage with straw incorporation; NTS, no tillage with straw mulching. SE, standard error.
