# Supplementary material for: Conservation tillage increases carbon sequestration of winter wheat-summer maize farmland on Loess Plateau in China
Source: PLoS One. 2018 Sep 5;13(9):e0199846. doi: 10.1371/journal.pone.0199846 (PMC6124710; doi:10.1371/journal.pone.0199846)
Supplement: S2 Table — (DOCX) [file pone.0199846.s002.docx]

**S2 Table. Soil microbial respiration under different tillage treatments (****mg CO_2_ m^−2^ h^−1^).**

| Sampling date | NTS | SE | RTS | SE | STS | SE | CT | SE |
| --- | --- | --- | --- | --- | --- | --- | --- | --- |
| 2013/10/20 |  |  |  |  |  |  |  |  |
| 2013/10/27 |  |  |  |  |  |  |  |  |
| 2013/11/4 | 110.6 | 9.28 | 269.8 | 24.51 | 300.9 | 34.85 | 257.7 | 29.86 |
| 2013/11/11 | 92.0 | 7.81 | 170.0 | 19.31 | 183.6 | 18.61 | 164.9 | 19.61 |
| 2013/11/18 | 107.0 | 9.08 | 222.3 | 18.54 | 227.5 | 23.80 | 193.2 | 23.88 |
| 2013/11/25 | 73.4 | 5.95 | 177.3 | 15.55 | 200.5 | 20.93 | 174.8 | 21.72 |
| 2013/12/1 | 66.1 | 6.88 | 163.3 | 14.19 | 182.7 | 19.13 | 158.1 | 20.83 |
| 2013/12/8 | 56.0 | 4.28 | 136.1 | 10.88 | 155.1 | 16.04 | 127.9 | 12.30 |
| 2013/12/15 | 65.0 | 6.65 | 100.3 | 10.52 | 124.8 | 11.51 | 111.3 | 14.15 |
| 2013/12/22 | 70.4 | 5.40 | 141.3 | 13.31 | 149.4 | 15.00 | 137.4 | 15.23 |
| 2013/12/29 | 54.4 | 4.03 | 127.3 | 10.44 | 151.7 | 15.00 | 124.2 | 14.10 |
| 2014/1/5 | 63.5 | 4.96 | 133.7 | 5.25 | 139.2 | 14.54 | 120.9 | 13.31 |
| 2014/1/12 | 39.0 | 4.33 | 128.6 | 9.83 | 141.6 | 11.64 | 121.1 | 12.90 |
| 2014/1/19 | 39.2 | 3.77 | 108.1 | 10.30 | 83.1 | 8.36 | 66.8 | 7.29 |
| 2014/2/2 | 68.8 | 6.71 | 84.5 | 8.14 | 84.0 | 6.58 | 79.8 | 7.90 |
| 2014/2/8 | 72.2 | 5.37 | 144.0 | 16.67 | 161.8 | 13.60 | 132.6 | 13.28 |
| 2014/2/15 | 68.4 | 6.08 | 107.1 | 13.34 | 109.8 | 8.46 | 92.4 | 9.46 |
| 2014/2/24 | 82.3 | 7.62 | 140.7 | 16.17 | 149.3 | 10.13 | 129.2 | 13.16 |
| 2014/3/2 | 82.1 | 8.13 | 253.0 | 23.36 | 249.3 | 14.53 | 231.4 | 24.21 |
| 2014/3/14 | 77.0 | 5.60 | 125.7 | 9.85 | 142.4 | 3.73 | 119.3 | 11.28 |
| 2014/3/21 | 88.1 | 5.45 | 98.4 | 8.25 | 113.0 | 6.01 | 92.1 | 8.95 |
| 2014/3/28 | 175.3 | 12.94 | 132.2 | 7.12 | 142.6 | 7.97 | 124.0 | 10.99 |
| 2014/4/1 | 147.7 | 10.47 | 165.9 | 10.96 | 171.8 | 12.01 | 155.5 | 13.44 |
| 2014/4/13 | 85.3 | 0.92 | 81.0 | 1.00 | 71.2 | 3.08 | 66.0 | 2.63 |
| 2014/4/19 | 113.6 | 5.67 | 164.7 | 5.33 | 166.7 | 6.28 | 154.4 | 5.68 |
| 2014/4/27 | 161.1 | 8.56 | 293.7 | 10.29 | 289.9 | 16.57 | 284.0 | 14.24 |
| 2014/5/2 | 188.9 | 5.18 | 278.6 | 10.07 | 277.4 | 17.64 | 261.8 | 12.88 |
| 2014/5/9 | 108.5 | 6.94 | 212.5 | 12.32 | 211.2 | 13.05 | 195.8 | 11.45 |
| 2014/5/16 | 182.1 | 9.95 | 218.1 | 9.67 | 210.6 | 15.29 | 206.1 | 13.89 |
| 2014/5/26 | 224.9 | 14.64 | 293.1 | 17.10 | 285.2 | 12.62 | 276.0 | 21.66 |
| 2014/6/1 | 244.4 | 18.29 | 248.7 | 15.07 | 239.6 | 9.73 | 245.5 | 23.03 |
| 2014/6/18 |  |  |  |  |  |  |  |  |
| 2014/6/21 |  |  |  |  |  |  |  |  |
| 2014/6/27 |  |  |  |  |  |  |  |  |
| 2014/6/30 | 174.8 | 15.69 | 219.2 | 18.44 | 224.9 | 26.81 | 253.5 | 39.69 |
| 2014/7/3 | 189.6 | 17.58 | 221.4 | 18.87 | 221.2 | 26.47 | 231.1 | 30.40 |
| 2014/7/6 | 83.5 | 9.94 | 129.0 | 12.59 | 119.4 | 6.55 | 105.8 | 16.73 |
| 2014/7/13 | 126.3 | 14.83 | 148.6 | 22.25 | 115.4 | 5.30 | 106.6 | 13.57 |
| 2014/7/16 | 52.6 | 5.08 | 151.2 | 13.83 | 142.4 | 7.46 | 89.4 | 11.32 |
| 2014/7/18 | 130.6 | 10.39 | 147.3 | 16.38 | 146.6 | 7.22 | 147.2 | 16.45 |
| 2014/7/21 | 124.0 | 13.36 | 158.3 | 18.11 | 155.6 | 8.27 | 161.2 | 18.28 |
| 2014/8/10 | 95.6 | 28.30 | 107.9 | 38.08 | 119.8 | 45.26 | 158.3 | 56.49 |
| 2014/8/14 | 129.6 | 14.75 | 228.5 | 26.24 | 174.1 | 14.42 | 156.5 | 39.42 |
| 2014/8/18 | 164.0 | 15.36 | 283.1 | 27.35 | 111.9 | 21.50 | 116.1 | 39.04 |
| 2014/8/23 | 89.1 | 9.13 | 173.0 | 21.40 | 138.6 | 11.94 | 127.2 | 24.33 |
| 2014/8/26 | 77.1 | 7.07 | 243.2 | 24.48 | 106.7 | 12.12 | 105.0 | 21.04 |
| 2014/9/5 | 224.6 | 21.70 | 280.1 | 28.98 | 297.3 | 24.24 | 261.9 | 23.45 |
| 2014/9/18 | 212.0 | 15.46 | 284.5 | 28.56 | 249.8 | 18.44 | 212.3 | 21.16 |
| 2014/9/21 | 95.8 | 12.96 | 289.6 | 22.63 | 209.7 | 16.12 | 211.0 | 11.36 |
| 2014/9/24 | 91.0 | 8.56 | 215.8 | 20.93 | 153.4 | 8.35 | 153.9 | 15.40 |
| 2014/9/25 | 136.2 | 16.20 | 261.9 | 24.26 | 194.8 | 8.34 | 187.1 | 18.46 |
| 2014/10/2 | 247.1 | 23.00 | 417.0 | 35.78 | 248.5 | 10.89 | 295.1 | 31.76 |
| 2014/10/5 | 74.0 | 8.93 | 153.9 | 15.51 | 129.4 | 5.32 | 86.8 | 9.10 |
| 2014/10/8 | 124.4 | 12.58 | 200.6 | 15.93 | 145.3 | 5.62 | 133.0 | 12.33 |
| 2014/10/11 | 91.3 | 7.99 | 163.9 | 14.99 | 122.1 | 4.82 | 114.2 | 8.57 |
| 2014/10/20 |  |  |  |  |  |  |  |  |
| 2014/10/27 |  |  |  |  |  |  |  |  |
| 2014/11/4 | 242.0 | 20.07 | 237.7 | 19.79 | 261.1 | 25.16 | 249.7 | 22.66 |
| 2014/11/11 | 99.2 | 9.24 | 201.6 | 16.48 | 220.9 | 20.54 | 207.1 | 18.61 |
| 2014/11/18 | 89.3 | 8.79 | 202.4 | 16.38 | 211.1 | 19.37 | 211.8 | 18.99 |
| 2014/11/25 | 71.6 | 8.06 | 171.7 | 13.49 | 176.7 | 15.63 | 177.5 | 15.87 |
| 2014/12/1 | 50.7 | 7.75 | 125.8 | 10.28 | 121.8 | 10.26 | 127.9 | 11.46 |
| 2014/12/8 | 48.5 | 7.64 | 121.9 | 9.94 | 131.0 | 10.88 | 126.4 | 11.46 |
| 2014/12/15 | 50.5 | 7.55 | 103.4 | 8.82 | 108.4 | 8.76 | 107.3 | 10.05 |
| 2014/12/22 | 46.0 | 7.53 | 127.9 | 9.92 | 137.7 | 9.98 | 122.7 | 9.41 |
| 2014/12/29 | 42.5 | 7.50 | 112.1 | 8.99 | 114.7 | 8.89 | 115.1 | 9.10 |
| 2015/1/5 | 54.4 | 7.45 | 116.4 | 9.16 | 128.9 | 9.38 | 125.1 | 9.50 |
| 2015/1/12 | 30.3 | 7.55 | 154.5 | 11.32 | 150.1 | 14.11 | 120.6 | 9.02 |
| 2015/1/19 | 39.7 | 7.41 | 73.8 | 7.44 | 68.9 | 6.10 | 67.2 | 5.00 |
| 2015/2/2 | 57.4 | 7.31 | 78.0 | 7.45 | 98.4 | 8.39 | 81.5 | 5.58 |
| 2015/2/8 | 48.4 | 7.24 | 138.8 | 10.10 | 153.5 | 14.20 | 134.1 | 9.47 |
| 2015/2/15 | 63.4 | 7.32 | 95.9 | 7.78 | 111.1 | 9.50 | 92.5 | 7.56 |
| 2015/2/24 | 72.5 | 7.37 | 138.4 | 9.65 | 133.9 | 11.81 | 133.1 | 9.30 |
| 2015/3/2 | 77.3 | 7.39 | 249.4 | 16.60 | 223.2 | 22.04 | 212.7 | 14.12 |
| 2015/3/14 | 66.5 | 6.75 | 127.3 | 8.69 | 118.4 | 9.48 | 120.2 | 6.88 |
| 2015/3/21 | 74.5 | 6.78 | 93.0 | 7.31 | 86.3 | 6.12 | 84.4 | 4.66 |
| 2015/3/28 | 209.6 | 12.48 | 159.9 | 10.29 | 212.5 | 9.76 | 147.7 | 8.21 |
| 2015/4/1 | 186.6 | 10.85 | 219.9 | 13.65 | 223.4 | 17.46 | 224.9 | 11.47 |
| 2015/4/13 | 80.3 | 5.25 | 123.5 | 5.36 | 103.3 | 2.36 | 94.5 | 3.21 |
| 2015/4/19 | 88.8 | 5.44 | 159.0 | 6.11 | 158.9 | 9.18 | 120.1 | 5.37 |
| 2015/4/27 | 153.3 | 6.84 | 348.5 | 13.78 | 274.7 | 19.49 | 265.7 | 9.56 |
| 2015/5/2 | 190.6 | 8.33 | 312.8 | 13.02 | 283.8 | 20.40 | 259.1 | 8.94 |
| 2015/5/9 | 102.6 | 5.90 | 150.5 | 6.95 | 199.6 | 14.06 | 149.8 | 7.61 |
| 2015/5/16 | 169.3 | 7.92 | 213.1 | 9.04 | 213.2 | 15.96 | 224.5 | 11.87 |
| 2015/5/26 | 248.0 | 12.15 | 283.8 | 12.90 | 265.6 | 11.99 | 303.4 | 19.07 |
| 2015/6/1 | 205.2 | 11.32 | 245.3 | 13.01 | 275.2 | 15.19 | 281.8 | 19.35 |
| 2015/6/18 |  |  |  |  |  |  |  |  |
| 2015/6/21 |  |  |  |  |  |  |  |  |
| 2015/6/27 |  |  |  |  |  |  |  |  |
| 2015/6/30 | 289.7 | 20.35 | 302.8 | 19.92 | 288.2 | 30.12 | 233.1 | 17.85 |
| 2015/7/3 | 119.5 | 8.94 | 209.0 | 16.66 | 228.3 | 23.35 | 182.9 | 13.64 |
| 2015/7/6 | 97.5 | 5.63 | 177.3 | 4.92 | 121.3 | 5.68 | 92.0 | 4.88 |
| 2015/7/13 | 62.1 | 5.08 | 137.3 | 2.15 | 78.4 | 5.85 | 80.6 | 5.15 |
| 2015/7/16 | 116.0 | 5.17 | 176.1 | 1.40 | 103.0 | 8.34 | 150.1 | 6.09 |
| 2015/7/18 | 148.6 | 5.40 | 208.8 | 1.95 | 138.9 | 7.33 | 156.4 | 6.55 |
| 2015/7/21 | 152.3 | 5.62 | 198.1 | 3.80 | 138.8 | 12.86 | 157.8 | 8.74 |
| 2015/8/10 | 94.7 | 24.46 | 115.5 | 37.28 | 83.4 | 43.15 | 135.9 | 38.34 |
| 2015/8/14 | 125.8 | 7.80 | 237.0 | 17.61 | 154.4 | 14.29 | 161.2 | 22.37 |
| 2015/8/18 | 170.6 | 5.73 | 288.5 | 12.08 | 84.3 | 25.23 | 101.6 | 35.29 |
| 2015/8/23 | 87.4 | 6.00 | 210.4 | 12.66 | 117.0 | 13.26 | 136.4 | 16.13 |
| 2015/8/26 | 77.5 | 5.09 | 230.0 | 4.75 | 96.9 | 13.08 | 99.9 | 18.60 |
| 2015/9/5 | 182.6 | 8.84 | 236.8 | 13.62 | 251.0 | 17.63 | 251.0 | 6.09 |
| 2015/9/18 | 179.1 | 5.45 | 295.1 | 5.18 | 189.5 | 12.18 | 208.6 | 8.25 |
| 2015/9/21 | 99.1 | 5.21 | 252.0 | 8.31 | 212.7 | 14.15 | 205.4 | 7.31 |
| 2015/9/24 | 100.3 | 5.13 | 206.4 | 1.63 | 134.7 | 8.26 | 156.3 | 8.51 |
| 2015/9/25 | 136.3 | 6.09 | 259.1 | 4.87 | 194.3 | 6.92 | 177.3 | 9.08 |
| 2015/10/2 | 139.4 | 5.39 | 191.8 | 3.15 | 195.3 | 21.01 | 248.2 | 11.84 |
| 2015/10/5 | 73.7 | 5.10 | 104.5 | 3.89 | 145.8 | 8.71 | 103.1 | 6.79 |
| 2015/10/8 | 152.8 | 5.38 | 153.4 | 1.78 | 153.3 | 10.21 | 146.5 | 6.06 |
| 2015/10/11 | 117.6 | 5.29 | 124.1 | 3.93 | 150.9 | 7.02 | 114.0 | 5.91 |

CT, conventional moldboard plowing tillage without crop straw; RTS, rotary tillage with straw incorporation; STS, chisel plow tillage with straw incorporation; NTS, no tillage with straw mulching. SE, standard error.
