# Supplementary material for: Conservation tillage increases carbon sequestration of winter wheat-summer maize farmland on Loess Plateau in China
Source: PLoS One. 2018 Sep 5;13(9):e0199846. doi: 10.1371/journal.pone.0199846 (PMC6124710; doi:10.1371/journal.pone.0199846)
Supplement: S3 Table — (DOCX) [file pone.0199846.s003.docx]

**S3 Table. Root Respiration under different tillage treatments (****mg CO_2_ m^−2^ h^−1^).**

| **Sampling Date** | **NTS** | **SE** | **RTS** | **SE** | **STS** | **SE** | **CT** | **SE** |
| --- | --- | --- | --- | --- | --- | --- | --- | --- |
| 2013/10/20 |  |  |  |  |  |  |  |  |
| 2013/10/27 |  |  |  |  |  |  |  |  |
| 2013/11/4 | 5.0 | 0.86 | 13.6 | 1.82 | 23.6 | 8.39 | 17.7 | 3.02 |
| 2013/11/11 | 6.1 | 1.04 | 12.3 | 1.95 | 16.7 | 5.44 | 13.3 | 2.40 |
| 2013/11/18 | 8.6 | 1.63 | 20.7 | 3.13 | 21.2 | 6.54 | 17.4 | 3.30 |
| 2013/11/25 | 7.2 | 1.34 | 20.7 | 3.34 | 25.1 | 5.45 | 19.6 | 3.90 |
| 2013/12/1 | 7.9 | 1.69 | 19.9 | 3.23 | 23.8 | 4.91 | 20.1 | 4.24 |
| 2013/12/8 | 7.1 | 1.26 | 19.2 | 3.09 | 23.5 | 4.13 | 17.9 | 3.22 |
| 2013/12/15 | 8.9 | 1.92 | 15.3 | 2.84 | 20.8 | 4.04 | 18.3 | 3.94 |
| 2013/12/22 | 10.2 | 1.90 | 24.1 | 4.35 | 29.2 | 4.12 | 24.6 | 5.00 |
| 2013/12/29 | 8.8 | 1.60 | 20.7 | 3.47 | 27.9 | 4.26 | 21.6 | 4.40 |
| 2014/1/5 | 10.4 | 1.99 | 23.0 | 3.41 | 28.6 | 3.65 | 23.9 | 4.91 |
| 2014/1/12 | 6.6 | 1.50 | 23.6 | 4.52 | 31.8 | 6.34 | 24.7 | 5.58 |
| 2014/1/19 | 6.4 | 1.36 | 17.3 | 2.18 | 19.2 | 3.19 | 14.2 | 3.26 |
| 2014/2/2 | 11.8 | 2.52 | 14.2 | 1.73 | 19.4 | 4.46 | 17.4 | 3.83 |
| 2014/2/8 | 12.5 | 2.34 | 23.8 | 2.50 | 38.5 | 7.21 | 29.4 | 6.50 |
| 2014/2/15 | 12.3 | 2.53 | 17.4 | 1.74 | 26.9 | 5.47 | 21.0 | 4.18 |
| 2014/2/24 | 15.8 | 3.34 | 25.0 | 2.65 | 38.3 | 6.69 | 32.8 | 6.64 |
| 2014/3/2 | 16.0 | 3.49 | 45.7 | 5.68 | 65.2 | 11.93 | 60.2 | 12.42 |
| 2014/3/14 | 25.2 | 5.16 | 43.2 | 5.64 | 55.2 | 9.04 | 45.5 | 9.65 |
| 2014/3/21 | 27.5 | 4.91 | 35.8 | 4.71 | 47.4 | 8.56 | 37.5 | 8.15 |
| 2014/3/28 | 62.8 | 13.12 | 53.4 | 10.01 | 63.0 | 11.03 | 54.2 | 11.53 |
| 2014/4/1 | 55.4 | 11.50 | 68.5 | 14.12 | 82.8 | 13.91 | 71.3 | 15.18 |
| 2014/4/13 | 82.8 | 17.03 | 84.4 | 16.49 | 85.5 | 14.64 | 63.6 | 13.90 |
| 2014/4/19 | 72.0 | 15.24 | 111.8 | 20.81 | 133.8 | 22.46 | 121.0 | 25.39 |
| 2014/4/27 | 94.5 | 19.89 | 192.8 | 36.27 | 216.8 | 36.54 | 194.2 | 42.01 |
| 2014/5/2 | 101.6 | 17.56 | 176.0 | 32.11 | 199.1 | 33.62 | 181.2 | 37.39 |
| 2014/5/9 | 59.6 | 12.94 | 112.0 | 22.36 | 123.3 | 20.67 | 114.8 | 23.52 |
| 2014/5/16 | 94.0 | 19.28 | 116.4 | 21.23 | 116.1 | 18.60 | 104.5 | 21.53 |
| 2014/5/26 | 94.6 | 19.50 | 120.1 | 22.41 | 130.4 | 24.31 | 115.6 | 24.05 |
| 2014/6/1 | 87.2 | 18.27 | 90.5 | 17.54 | 97.1 | 17.12 | 65.1 | 13.38 |
| 2014/6/18 |  |  |  |  |  |  |  |  |
| 2014/6/21 |  |  |  |  |  |  |  |  |
| 2014/6/27 |  |  |  |  |  |  |  |  |
| 2014/6/30 | 35.3 | 6.50 | 59.7 | 7.67 | 44.1 | 4.90 | 46.5 | 11.09 |
| 2014/7/3 | 34.5 | 6.38 | 62.9 | 8.55 | 41.8 | 4.78 | 45.9 | 9.34 |
| 2014/7/6 | 79.8 | 14.43 | 124.0 | 19.01 | 157.6 | 23.24 | 110.2 | 9.61 |
| 2014/7/13 | 109.9 | 20.41 | 202.4 | 30.87 | 230.6 | 35.61 | 153.4 | 27.66 |
| 2014/7/16 | 45.5 | 7.85 | 172.8 | 27.58 | 196.6 | 29.17 | 113.5 | 21.11 |
| 2014/7/18 | 92.6 | 17.14 | 178.3 | 31.65 | 215.4 | 32.21 | 202.8 | 28.95 |
| 2014/7/21 | 118.3 | 22.00 | 206.2 | 35.31 | 240.4 | 47.78 | 205.8 | 32.74 |
| 2014/8/10 | 300.5 | 57.28 | 449.9 | 78.71 | 578.9 | 115.05 | 538.0 | 101.08 |
| 2014/8/14 | 162.7 | 30.59 | 310.4 | 55.04 | 418.9 | 63.46 | 371.7 | 56.11 |
| 2014/8/18 | 158.4 | 29.93 | 277.3 | 51.20 | 500.1 | 78.60 | 434.3 | 113.02 |
| 2014/8/23 | 107.0 | 19.63 | 261.5 | 45.67 | 346.8 | 52.70 | 308.0 | 63.94 |
| 2014/8/26 | 68.1 | 12.19 | 231.1 | 38.83 | 332.0 | 51.21 | 277.2 | 50.86 |
| 2014/9/5 | 260.1 | 49.95 | 295.1 | 45.22 | 359.4 | 50.45 | 314.1 | 59.82 |
| 2014/9/18 | 155.5 | 29.54 | 267.8 | 41.66 | 383.6 | 55.61 | 299.7 | 53.41 |
| 2014/9/21 | 91.6 | 16.74 | 231.7 | 38.58 | 288.9 | 41.31 | 249.0 | 40.74 |
| 2014/9/24 | 73.8 | 13.35 | 177.7 | 32.75 | 238.9 | 47.49 | 226.1 | 42.47 |
| 2014/9/25 | 79.4 | 14.65 | 224.6 | 32.06 | 236.5 | 38.61 | 224.9 | 38.13 |
| 2014/10/2 | 225.6 | 43.29 | 272.4 | 46.78 | 435.8 | 73.11 | 402.9 | 71.33 |
| 2014/10/5 | 65.3 | 11.64 | 129.3 | 23.60 | 163.6 | 26.80 | 110.2 | 20.58 |
| 2014/10/8 | 99.8 | 18.47 | 175.9 | 33.79 | 197.9 | 32.61 | 147.0 | 25.89 |
| 2014/10/11 | 69.8 | 12.60 | 140.4 | 23.81 | 161.4 | 26.55 | 119.4 | 25.30 |
| 2014/10/20 |  |  |  |  |  |  |  |  |
| 2014/10/27 |  |  |  |  |  |  |  |  |
| 2014/11/4 | 12.0 | 2.16 | 16.3 | 2.38 | 23.9 | 6.10 | 20.6 | 1.00 |
| 2014/11/11 | 7.8 | 1.31 | 16.4 | 2.45 | 23.1 | 5.32 | 19.4 | 1.07 |
| 2014/11/18 | 7.5 | 1.25 | 18.6 | 2.84 | 23.9 | 5.17 | 22.2 | 1.38 |
| 2014/11/25 | 7.8 | 1.31 | 21.3 | 3.33 | 22.3 | 4.58 | 21.1 | 1.46 |
| 2014/12/1 | 6.0 | 1.10 | 16.2 | 2.50 | 17.2 | 3.60 | 17.1 | 1.20 |
| 2014/12/8 | 6.5 | 1.21 | 17.2 | 2.66 | 21.0 | 3.97 | 19.6 | 1.48 |
| 2014/12/15 | 7.5 | 1.36 | 15.7 | 2.63 | 20.6 | 3.78 | 19.7 | 1.59 |
| 2014/12/22 | 7.0 | 1.32 | 21.1 | 3.61 | 29.2 | 5.05 | 23.1 | 3.95 |
| 2014/12/29 | 6.7 | 1.33 | 19.9 | 3.37 | 23.3 | 3.96 | 20.9 | 3.55 |
| 2015/1/5 | 9.0 | 1.59 | 21.6 | 3.41 | 29.0 | 5.00 | 23.9 | 4.09 |
| 2015/1/12 | 5.3 | 1.33 | 30.5 | 4.90 | 36.6 | 6.81 | 26.4 | 4.52 |
| 2015/1/19 | 7.1 | 1.43 | 14.2 | 2.28 | 18.0 | 4.23 | 16.4 | 2.91 |
| 2015/2/2 | 11.0 | 1.89 | 16.0 | 2.56 | 26.6 | 5.28 | 20.5 | 3.36 |
| 2015/2/8 | 9.6 | 1.74 | 30.2 | 4.86 | 44.1 | 7.33 | 33.9 | 5.50 |
| 2015/2/15 | 12.6 | 2.13 | 23.1 | 3.67 | 32.9 | 5.92 | 23.5 | 3.75 |
| 2015/2/24 | 15.5 | 2.60 | 35.6 | 5.80 | 42.1 | 6.92 | 35.6 | 5.79 |
| 2015/3/2 | 17.5 | 2.92 | 61.6 | 11.26 | 71.6 | 10.35 | 58.3 | 9.83 |
| 2015/3/14 | 20.4 | 3.41 | 36.7 | 6.34 | 50.7 | 7.59 | 46.6 | 8.11 |
| 2015/3/21 | 23.9 | 3.98 | 30.0 | 4.84 | 41.8 | 7.06 | 34.6 | 6.18 |
| 2015/3/28 | 69.6 | 12.62 | 50.7 | 8.62 | 107.5 | 19.68 | 62.9 | 11.12 |
| 2015/4/1 | 66.4 | 11.93 | 71.7 | 12.56 | 124.9 | 15.37 | 99.1 | 18.73 |
| 2015/4/13 | 85.5 | 14.77 | 124.7 | 21.96 | 132.5 | 18.43 | 83.7 | 15.24 |
| 2015/4/19 | 59.2 | 10.12 | 116.0 | 20.54 | 130.1 | 17.14 | 89.9 | 14.99 |
| 2015/4/27 | 89.6 | 16.06 | 210.7 | 38.98 | 202.1 | 26.19 | 177.3 | 31.26 |
| 2015/5/2 | 104.4 | 19.01 | 181.2 | 33.28 | 206.3 | 26.67 | 177.9 | 31.40 |
| 2015/5/9 | 59.4 | 10.24 | 90.1 | 15.73 | 132.4 | 16.83 | 76.0 | 12.68 |
| 2015/5/16 | 88.7 | 15.97 | 117.8 | 21.63 | 128.9 | 16.10 | 102.5 | 17.48 |
| 2015/5/26 | 114.0 | 21.05 | 142.3 | 26.55 | 134.4 | 24.98 | 105.6 | 17.97 |
| 2015/6/1 | 79.6 | 14.45 | 100.5 | 18.51 | 105.8 | 19.60 | 64.0 | 11.78 |
| 2015/6/18 |  |  |  |  |  |  |  |  |
| 2015/6/21 |  |  |  |  |  |  |  |  |
| 2015/6/27 |  |  |  |  |  |  |  |  |
| 2015/6/30 | 60.6 | 11.17 | 69.6 | 11.40 | 65.3 | 10.70 | 45.9 | 6.64 |
| 2015/7/3 | 26.5 | 4.53 | 50.7 | 9.10 | 51.1 | 8.56 | 38.1 | 5.53 |
| 2015/7/6 | 61.2 | 10.54 | 125.9 | 22.92 | 173.3 | 27.02 | 129.0 | 21.54 |
| 2015/7/13 | 53.9 | 9.05 | 125.7 | 23.81 | 174.6 | 27.78 | 121.4 | 20.15 |
| 2015/7/16 | 99.8 | 17.68 | 155.9 | 27.97 | 237.0 | 38.03 | 215.9 | 37.40 |
| 2015/7/18 | 119.4 | 21.56 | 175.2 | 30.52 | 232.1 | 36.68 | 229.6 | 39.92 |
| 2015/7/21 | 156.6 | 28.59 | 209.9 | 36.37 | 260.2 | 49.95 | 215.2 | 39.83 |
| 2015/8/10 | 333.1 | 62.17 | 475.5 | 79.31 | 518.6 | 100.71 | 516.1 | 98.35 |
| 2015/8/14 | 173.2 | 31.55 | 361.0 | 59.55 | 427.4 | 64.37 | 372.8 | 70.41 |
| 2015/8/18 | 175.4 | 32.30 | 319.5 | 54.64 | 519.7 | 81.22 | 452.4 | 85.59 |
| 2015/8/23 | 110.6 | 19.45 | 284.6 | 49.91 | 372.0 | 56.35 | 301.6 | 54.94 |
| 2015/8/26 | 71.5 | 12.24 | 234.0 | 39.94 | 345.1 | 52.50 | 292.1 | 52.96 |
| 2015/9/5 | 239.4 | 44.65 | 321.2 | 53.68 | 332.0 | 46.70 | 300.0 | 54.89 |
| 2015/9/18 | 152.9 | 28.08 | 264.9 | 43.94 | 361.5 | 52.86 | 284.4 | 51.87 |
| 2015/9/21 | 96.9 | 17.02 | 279.0 | 46.78 | 304.3 | 43.16 | 268.6 | 48.86 |
| 2015/9/24 | 87.7 | 15.33 | 191.6 | 33.47 | 259.3 | 37.76 | 232.7 | 41.92 |
| 2015/9/25 | 86.7 | 15.42 | 174.9 | 29.88 | 248.7 | 47.92 | 237.7 | 44.28 |
| 2015/10/2 | 137.6 | 24.92 | 200.2 | 35.13 | 400.7 | 77.79 | 338.8 | 64.27 |
| 2015/10/5 | 62.3 | 10.57 | 93.5 | 19.29 | 222.2 | 42.53 | 137.9 | 24.68 |
| 2015/10/8 | 125.2 | 22.68 | 131.6 | 25.45 | 245.7 | 47.17 | 162.5 | 29.69 |
| 2015/10/11 | 91.4 | 16.15 | 99.9 | 20.50 | 208.1 | 39.80 | 131.0 | 23.48 |

CT, conventional moldboard plowing tillage without crop straw; RTS, rotary tillage with straw incorporation; STS, chisel plow tillage with straw incorporation; NTS, no tillage with straw mulching. SE, standard error.
