# Supplementary material for: Conservation tillage increases carbon sequestration of winter wheat-summer maize farmland on Loess Plateau in China
Source: PLoS One. 2018 Sep 5;13(9):e0199846. doi: 10.1371/journal.pone.0199846 (PMC6124710; doi:10.1371/journal.pone.0199846)
Supplement: S4 Table — (DOCX) [file pone.0199846.s004.docx]

**S4 Table. The ratio of root respiration to total soil respiration under different tillage treatments (%).**

| **Sampling date** | **NTS** | **SE** | **RTS** | **SE** | **STS** | **SE** | **CT** | **SE** |
| --- | --- | --- | --- | --- | --- | --- | --- | --- |
| 2013/10/20 |  |  |  |  |  |  |  |  |
| 2013/10/27 |  |  |  |  |  |  |  |  |
| 2013/11/4 | 4.3 | 0.4 | 4.8 | 0.3 | 7.4 | 2.8 | 6.4 | 0.4 |
| 2013/11/11 | 6.2 | 0.6 | 6.7 | 0.4 | 8.4 | 2.8 | 7.4 | 0.5 |
| 2013/11/18 | 7.4 | 0.7 | 8.5 | 0.6 | 8.6 | 2.8 | 8.2 | 0.5 |
| 2013/11/25 | 8.9 | 0.9 | 10.4 | 0.7 | 11.2 | 2.6 | 10.0 | 0.7 |
| 2013/12/1 | 10.5 | 1.1 | 10.8 | 0.8 | 11.6 | 2.5 | 11.2 | 0.8 |
| 2013/12/8 | 11.2 | 1.1 | 12.3 | 0.9 | 13.2 | 2.4 | 12.2 | 0.9 |
| 2013/12/15 | 12.0 | 1.2 | 13.2 | 1.0 | 14.3 | 2.3 | 14.0 | 1.1 |
| 2013/12/22 | 12.6 | 1.3 | 14.5 | 1.1 | 16.4 | 2.2 | 15.1 | 1.2 |
| 2013/12/29 | 13.8 | 1.4 | 13.9 | 1.1 | 15.6 | 2.3 | 14.7 | 1.2 |
| 2014/1/5 | 14.0 | 1.4 | 14.6 | 1.5 | 17.1 | 2.2 | 16.4 | 1.4 |
| 2014/1/12 | 14.4 | 1.4 | 15.4 | 1.5 | 18.3 | 2.7 | 16.8 | 1.7 |
| 2014/1/19 | 14.0 | 1.4 | 13.8 | 1.4 | 18.8 | 2.7 | 17.4 | 1.7 |
| 2014/2/2 | 14.5 | 1.5 | 14.4 | 1.4 | 18.6 | 2.7 | 17.8 | 1.8 |
| 2014/2/8 | 14.7 | 1.5 | 14.2 | 1.4 | 19.2 | 2.7 | 18.0 | 1.8 |
| 2014/2/15 | 15.1 | 1.5 | 14.0 | 1.4 | 19.6 | 2.7 | 18.4 | 1.5 |
| 2014/2/24 | 16.0 | 1.6 | 15.1 | 1.4 | 20.3 | 2.0 | 20.1 | 1.7 |
| 2014/3/2 | 16.2 | 1.6 | 15.3 | 1.4 | 20.6 | 2.1 | 20.5 | 1.7 |
| 2014/3/14 | 24.5 | 2.5 | 25.5 | 1.4 | 27.8 | 2.8 | 27.4 | 2.4 |
| 2014/3/21 | 23.7 | 2.4 | 26.6 | 1.5 | 29.4 | 2.9 | 28.7 | 2.5 |
| 2014/3/28 | 26.2 | 2.6 | 28.6 | 2.9 | 30.5 | 3.0 | 30.2 | 2.7 |
| 2014/4/1 | 27.1 | 2.7 | 29.0 | 2.9 | 32.4 | 3.1 | 31.2 | 2.8 |
| 2014/4/13 | 48.9 | 4.9 | 50.7 | 5.1 | 54.3 | 4.7 | 48.7 | 4.5 |
| 2014/4/19 | 38.5 | 3.9 | 40.2 | 3.8 | 44.3 | 3.9 | 43.6 | 4.4 |
| 2014/4/27 | 36.7 | 3.7 | 39.4 | 3.7 | 42.6 | 3.8 | 40.3 | 4.0 |
| 2014/5/2 | 34.8 | 3.5 | 38.5 | 3.7 | 41.6 | 3.7 | 40.6 | 3.9 |
| 2014/5/9 | 35.2 | 3.5 | 34.3 | 3.2 | 36.7 | 3.4 | 36.7 | 3.5 |
| 2014/5/16 | 33.8 | 3.4 | 34.6 | 3.3 | 35.4 | 3.3 | 33.4 | 3.1 |
| 2014/5/26 | 29.4 | 2.9 | 28.9 | 2.7 | 31.2 | 3.1 | 29.3 | 2.7 |
| 2014/6/1 | 26.1 | 2.6 | 26.5 | 2.7 | 28.7 | 2.9 | 20.8 | 1.9 |
| 2014/6/18 |  |  |  |  |  |  |  |  |
| 2014/6/21 |  |  |  |  |  |  |  |  |
| 2014/6/27 |  |  |  |  |  |  |  |  |
| 2014/6/30 | 16.7 | 1.7 | 21.4 | 1.5 | 16.5 | 2.2 | 15.4 | 1.2 |
| 2014/7/3 | 15.3 | 1.5 | 22.1 | 1.6 | 16.0 | 2.2 | 16.5 | 1.3 |
| 2014/7/6 | 48.7 | 4.9 | 48.9 | 4.2 | 56.7 | 3.2 | 51.2 | 3.6 |
| 2014/7/13 | 46.3 | 4.6 | 57.6 | 5.1 | 66.4 | 4.0 | 58.8 | 5.7 |
| 2014/7/16 | 46.2 | 4.6 | 53.2 | 4.6 | 57.8 | 3.3 | 55.7 | 5.8 |
| 2014/7/18 | 41.3 | 4.1 | 54.6 | 4.8 | 59.3 | 3.4 | 57.9 | 3.8 |
| 2014/7/21 | 48.6 | 4.9 | 56.4 | 5.0 | 60.3 | 6.0 | 56.0 | 4.7 |
| 2014/8/10 | 75.6 | 7.6 | 80.4 | 7.4 | 82.3 | 8.2 | 77.0 | 8.6 |
| 2014/8/14 | 55.4 | 5.5 | 57.4 | 5.7 | 70.4 | 4.4 | 70.3 | 7.0 |
| 2014/8/18 | 48.9 | 4.9 | 49.3 | 4.9 | 81.4 | 5.3 | 78.4 | 7.8 |
| 2014/8/23 | 54.3 | 5.4 | 60.0 | 5.5 | 71.2 | 4.5 | 70.4 | 7.0 |
| 2014/8/26 | 46.7 | 4.7 | 48.6 | 4.4 | 75.4 | 4.8 | 72.2 | 6.9 |
| 2014/9/5 | 53.4 | 5.3 | 51.2 | 4.6 | 54.6 | 3.3 | 54.3 | 5.3 |
| 2014/9/18 | 42.1 | 4.2 | 48.4 | 4.4 | 60.4 | 3.6 | 58.3 | 5.6 |
| 2014/9/21 | 48.7 | 4.9 | 44.3 | 4.0 | 57.8 | 3.5 | 53.9 | 4.0 |
| 2014/9/24 | 44.6 | 4.5 | 45.0 | 4.0 | 60.5 | 6.1 | 59.3 | 3.9 |
| 2014/9/25 | 36.7 | 3.7 | 46.1 | 4.1 | 54.6 | 3.9 | 54.4 | 5.2 |
| 2014/10/2 | 47.5 | 4.8 | 39.4 | 3.9 | 63.4 | 4.7 | 57.5 | 5.8 |
| 2014/10/5 | 46.7 | 4.7 | 45.5 | 4.6 | 55.6 | 4.0 | 55.7 | 5.6 |
| 2014/10/8 | 44.3 | 4.4 | 46.5 | 4.7 | 57.4 | 4.1 | 52.3 | 5.2 |
| 2014/10/11 | 43.1 | 4.3 | 46.0 | 4.6 | 56.7 | 4.1 | 50.8 | 5.1 |
| 2014/10/20 |  |  |  |  |  |  |  |  |
| 2014/10/27 |  |  |  |  |  |  |  |  |
| 2014/11/4 | 4.7 | 0.5 | 6.4 | 0.4 | 8.4 | 2.1 | 7.6 | 0.3 |
| 2014/11/11 | 7.3 | 0.7 | 7.5 | 0.5 | 9.5 | 2.0 | 8.6 | 0.3 |
| 2014/11/18 | 7.7 | 0.8 | 8.4 | 0.6 | 10.2 | 2.0 | 9.5 | 0.3 |
| 2014/11/25 | 9.8 | 1.0 | 11.0 | 0.9 | 11.2 | 1.9 | 10.6 | 0.3 |
| 2014/12/1 | 10.6 | 1.1 | 11.4 | 0.9 | 12.3 | 1.9 | 11.8 | 0.3 |
| 2014/12/8 | 11.8 | 1.2 | 12.3 | 1.0 | 13.8 | 1.8 | 13.4 | 0.3 |
| 2014/12/15 | 12.9 | 1.3 | 13.1 | 1.3 | 15.9 | 1.8 | 15.5 | 0.4 |
| 2014/12/22 | 13.2 | 1.3 | 14.1 | 1.4 | 17.4 | 1.7 | 15.8 | 1.6 |
| 2014/12/29 | 13.7 | 1.4 | 15.0 | 1.5 | 16.8 | 1.7 | 15.3 | 1.5 |
| 2015/1/5 | 14.2 | 1.4 | 15.6 | 1.3 | 18.3 | 1.8 | 16.0 | 1.6 |
| 2015/1/12 | 14.9 | 1.5 | 16.4 | 1.4 | 19.6 | 3.0 | 17.9 | 1.8 |
| 2015/1/19 | 15.1 | 1.5 | 16.1 | 1.3 | 20.6 | 3.0 | 19.5 | 1.7 |
| 2015/2/2 | 16.0 | 1.6 | 17.0 | 1.4 | 21.2 | 2.9 | 20.0 | 1.7 |
| 2015/2/8 | 16.5 | 1.7 | 17.8 | 1.5 | 22.3 | 2.9 | 20.1 | 1.7 |
| 2015/2/15 | 16.5 | 1.7 | 19.3 | 1.6 | 22.8 | 2.9 | 20.2 | 1.7 |
| 2015/2/24 | 17.6 | 1.8 | 20.4 | 1.7 | 23.9 | 2.8 | 21.0 | 1.8 |
| 2015/3/2 | 18.4 | 1.8 | 19.7 | 2.0 | 24.3 | 2.8 | 21.4 | 1.8 |
| 2015/3/14 | 23.4 | 2.3 | 22.3 | 2.2 | 29.9 | 2.6 | 27.8 | 2.5 |
| 2015/3/21 | 24.2 | 2.4 | 24.3 | 2.3 | 32.5 | 3.3 | 28.9 | 2.9 |
| 2015/3/28 | 24.8 | 2.5 | 24.0 | 2.2 | 33.4 | 3.3 | 29.7 | 3.0 |
| 2015/4/1 | 26.1 | 2.6 | 24.5 | 2.3 | 35.8 | 1.8 | 30.4 | 3.0 |
| 2015/4/13 | 51.3 | 5.1 | 50.0 | 4.8 | 56.0 | 3.1 | 46.7 | 4.7 |
| 2015/4/19 | 39.8 | 4.0 | 42.0 | 4.0 | 44.9 | 2.3 | 42.6 | 3.8 |
| 2015/4/27 | 36.7 | 3.7 | 37.5 | 3.6 | 42.3 | 2.1 | 39.8 | 3.5 |
| 2015/5/2 | 35.2 | 3.5 | 36.5 | 3.5 | 42.0 | 2.1 | 40.5 | 3.6 |
| 2015/5/9 | 36.5 | 3.7 | 37.3 | 3.6 | 39.8 | 2.0 | 33.5 | 2.9 |
| 2015/5/16 | 34.2 | 3.4 | 35.4 | 3.5 | 37.6 | 1.9 | 31.2 | 2.7 |
| 2015/5/26 | 31.3 | 3.1 | 33.2 | 3.3 | 33.4 | 3.3 | 25.7 | 2.1 |
| 2015/6/1 | 27.8 | 2.8 | 28.9 | 2.9 | 27.6 | 2.8 | 18.4 | 1.8 |
| 2015/6/18 |  |  |  |  |  |  |  |  |
| 2015/6/21 |  |  |  |  |  |  |  |  |
| 2015/6/27 |  |  |  |  |  |  |  |  |
| 2015/6/30 | 17.2 | 1.7 | 18.6 | 1.5 | 18.5 | 2.7 | 16.4 | 1.0 |
| 2015/7/3 | 18.1 | 1.8 | 19.4 | 1.6 | 18.3 | 2.7 | 17.2 | 1.1 |
| 2015/7/6 | 38.4 | 3.8 | 41.3 | 3.8 | 58.6 | 4.2 | 58.1 | 5.1 |
| 2015/7/13 | 46.3 | 4.6 | 47.5 | 4.4 | 68.7 | 5.1 | 59.8 | 5.2 |
| 2015/7/16 | 46.0 | 4.6 | 46.7 | 4.3 | 69.4 | 5.1 | 58.7 | 5.1 |
| 2015/7/18 | 44.3 | 4.4 | 45.4 | 4.2 | 62.3 | 4.5 | 59.2 | 5.2 |
| 2015/7/21 | 50.4 | 5.0 | 51.2 | 4.8 | 64.8 | 6.5 | 57.4 | 5.7 |
| 2015/8/10 | 77.4 | 7.7 | 80.1 | 7.7 | 85.6 | 8.6 | 78.7 | 7.9 |
| 2015/8/14 | 57.6 | 5.8 | 60.1 | 5.7 | 73.2 | 4.6 | 69.4 | 6.9 |
| 2015/8/18 | 50.4 | 5.0 | 52.3 | 5.2 | 85.7 | 5.7 | 81.2 | 8.1 |
| 2015/8/23 | 55.6 | 5.6 | 57.2 | 5.7 | 75.8 | 4.8 | 68.5 | 6.5 |
| 2015/8/26 | 47.8 | 4.8 | 50.2 | 4.7 | 77.8 | 5.0 | 74.1 | 7.1 |
| 2015/9/5 | 56.4 | 5.6 | 57.3 | 5.4 | 56.8 | 3.4 | 54.1 | 5.1 |
| 2015/9/18 | 45.8 | 4.6 | 47.1 | 4.4 | 65.4 | 4.0 | 57.4 | 5.4 |
| 2015/9/21 | 49.2 | 4.9 | 52.3 | 4.9 | 58.7 | 3.5 | 56.4 | 5.3 |
| 2015/9/24 | 46.4 | 4.6 | 47.9 | 4.5 | 65.6 | 4.0 | 59.5 | 5.6 |
| 2015/9/25 | 38.7 | 3.9 | 40.1 | 3.7 | 55.8 | 5.6 | 56.9 | 5.7 |
| 2015/10/2 | 49.4 | 4.9 | 50.8 | 4.8 | 66.8 | 6.7 | 57.4 | 5.7 |
| 2015/10/5 | 45.6 | 4.6 | 46.9 | 4.4 | 60.0 | 6.0 | 56.9 | 5.7 |
| 2015/10/8 | 44.8 | 4.5 | 45.9 | 4.6 | 61.2 | 6.1 | 52.3 | 5.2 |
| 2015/10/11 | 43.5 | 4.4 | 44.3 | 4.4 | 57.6 | 5.8 | 53.2 | 5.3 |

CT, conventional moldboard plowing tillage without crop straw; RTS, rotary tillage with straw incorporation; STS, chisel plow tillage with straw incorporation; NTS, no tillage with straw mulching. SE, standard error.
