# Supplementary material for: Conservation tillage increases carbon sequestration of winter wheat-summer maize farmland on Loess Plateau in China
Source: PLoS One. 2018 Sep 5;13(9):e0199846. doi: 10.1371/journal.pone.0199846 (PMC6124710; doi:10.1371/journal.pone.0199846)
Supplement: S5 Table — (DOCX) [file pone.0199846.s005.docx]

**S6 Table. The agro-ecosystem C balance (NCF) and its main components for the annual cycle of wheat-maize rotation in 2013-2015 (t C ha^-1^ yr^-1^).**

| **Year** | **Treatment** | **NPP** | **R_h_** | **Harvest** | **NECB** | **C_AP_** | **NCF** |
| --- | --- | --- | --- | --- | --- | --- | --- |
|  | NTS | 10.85±0.46bc | 2.72±0.07d | 4.95±0.25c | 3.12±0.28a | 0.59 | 2.53±0.28a |
| **2013-14** | RTS | 10.95±0.42b | 4.28±0.11a | 4.91±0.25c | 1.72±0.38b | 0.67 | 1.05±0.38b |
|  | STS | 11.71±0.46a | 4.12±0.14b | 5.53±0.28b | 1.91±0.29b | 0.67 | 1.24±0.29b |
|  | CT | 10.40±0.46c | 3.80±0.12c | 9.41±0.42a | -2.85±0.19c | 0.76 | -3.61±0.19c |
|  | NTS | 10.99±0.44b | 2.71±0.09c | 5.01±0.25c | 3.28±0.26a | 0.59 | 2.69±0.26a |
| **2014-15** | RTS | 10.84±0.21bc | 4.38±0.08a | 4.95±0.12c | 1.51±0.17c | 0.67 | 0.84±0.17c |
|  | STS | 12.02±0.58a | 3.95±0.10b | 5.68±0.27b | 2.39±0.37b | 0.67 | 1.72±0.37b |
|  | CT | 10.45±0.33c | 3.77±0.08b | 9.45±0.30a | -2.77±0.12d | 0.76 | -3.53±0.12d |

The different lowercase letters following the same column represent significant difference at 5% levels. CT: conventional moldboard plowing tillage without crop straw; RTS: rotary tillage with straw incorporation; STS: chisel plow tillage with straw incorporation; NTS: no tillage with straw mulching; NPP: net primary productivity; Rh: microbial respiration; NECB: net ecosystem carbon balance without considering the carbon emission from farm inputs; C_AP_: the carbon emission from agricultural input; NCF: the net ecosystem carbon balance with considering the carbon emission from farm inputs. The values following the symbols are the standard errors.
