# Supplementary material for: Conservation tillage increases carbon sequestration of winter wheat-summer maize farmland on Loess Plateau in China
Source: PLoS One. 2018 Sep 5;13(9):e0199846. doi: 10.1371/journal.pone.0199846 (PMC6124710; doi:10.1371/journal.pone.0199846)
Supplement: S6 Table — (DOCX) [file pone.0199846.s006.docx]

**S7 Table. The carbon productivity under different treatments.**

| **Treatments** | **Wheat season** | **Maize season** | **Annual** |
| --- | --- | --- | --- |
| **2013-14** |  |  |  |
| **STS** | 7.34±0.38a | 9.12±0.45a | 8.27±0.41a |
| **NTS** | 7.78±0.33a | 8.81±0.52a | 8.32±0.42a |
| **RTS** | 5.56±0.30b | 6.44±0.37b | 6.02±0.30b |
| **CT** | 5.72±0.26b | 6.72±0.36b | 6.24±0.27b |
| **2014-15** |  |  |  |
| **STS** | 7.69±0.39a | 9.21±0.41a | 8.49±0.40a |
| **NTS** | 7.90±0.38a | 8.88±0.50a | 8.42±0.42a |
| **RTS** | 5.55±0.27b | 6.54±0.33b | 6.07±0.15b |
| **CT** | 5.81±0.28b | 6.73±0.35b | 6.29±0.23b |

The different lowercase letters following the same column represent significant difference at 5% levels. CT, conventional moldboard plowing tillage without crop straw; RTS, rotary tillage with straw incorporation; STS, chisel plow tillage with straw incorporation; NTS, no tillage with straw mulching. The values following the symbols are the standard errors.
